# The therapeutic potential of sphingolipids for cardiovascular diseases

**DOI:** 10.3389/fcvm.2023.1224743

**Published:** 2023-08-07

**Authors:** Sapir Ya'ar Bar, Noam Pintel, Hesen Abd Alghne, Hamdan Khattib, Dorit Avni

**Affiliations:** ^1^ Department of Natural Compound, Nutrition, and Health, MIGAL, Kiryat Shmona, Israel; ^2^ Tel-Hai College Department of Biotechnology, Kiryat Shmona, Israel; ^3^ Department of Gastroenterology and Hepatology, Tel Aviv University Sackler Faculty of Medicine, Tel Aviv, Israel

**Keywords:** cardiovascular diseases, inflammation, sphingolipids, ceramide, macrophages

## Abstract

Cardiovascular diseases (CVDs) are the leading cause of morbidity and mortality worldwide and Inflammation plays a critical role in the development of CVD. Despite considerable progress in understanding the underlying mechanisms and various treatment options available, significant gaps in therapy necessitate the identification of novel therapeutic targets. Sphingolipids are a family of lipids that have gained attention in recent years as important players in CVDs and the inflammatory processes that underlie their development. As preclinical studies have shown that targeting sphingolipids can modulate inflammation and ameliorate CVDs, targeting sphingolipids has emerged as a promising therapeutic strategy. This review discusses the current understanding of sphingolipids’ involvement in inflammation and cardiovascular diseases, the existing therapeutic approaches and gaps in therapy, and explores the potential of sphingolipids-based drugs as a future avenue for CVD treatment.

## Introduction

Cardiovascular diseases (CVDs) are illnesses that affect the heart and blood vessels and are a major global health concern, representing the leading cause of death and disability worldwide (1). According to the World Health Organization (WHO), approximately 18 million people die each year due to CVDs, which account for 32% of all global deaths (2). This group includes disorders such as coronary artery disease (CAD), peripheral arterial disease (PAD), stroke, heart failure, cardiomyopathy and congenital heart disease (CHD). Despite substantial progress in understanding the pathophysiology and the development of various therapeutic approaches, they remain a main public health concern (3).

The development and progression of CVDs are complex, multifactorial processes that involve a combination of genetic, environmental, and lifestyle factors. Inflammation, oxidative stress, endothelial dysfunction, and dyslipidemia are some of the key mechanisms that contribute to the pathogenesis of CVDs (4). Inflammation plays a critical role in the development of CVD (5). Inflammation is a protective response involving the immune system. It is a biological reaction that can be activated by a range of factors, including infections, pathogens, or damaged cells, and can lead to acute or chronic inflammatory responses in many diseases, including cardiovascular disease (6). Recent studies suggest that targeting inflammation may offer a new approach to reducing the risk of acute CV events.

Sphingolipids are a family of complex lipids that are involved in various cellular processes, including proliferation, signal transduction, apoptosis and cell differentiation (7). Over the past decade, sphingolipids have been identified as a new class of bioactive lipids that play a critical role in the development and progression of CVDs.

Recent studies have shown that sphingolipids contribute to the pathophysiology of CVDs by regulating inflammation, endothelial function, and lipid metabolism (8). The sphingolipid pathway is also implicated in the development of atherosclerosis, myocardial infarction, heart failure, and other CVDs (9).

Inflammation, a key driver of CVDs, plays a pivotal role in the initiation and progression of atherosclerosis, plaque instability, and myocardial damage (10). Sphingolipids have emerged as potent modulators of inflammatory pathways involved in the pathogenesis of CVDs. These bioactive lipids can regulate the expression of pro-inflammatory mediators, such as cytokines and adhesion molecules, and activate signaling pathways involved in immune cell recruitment and activation (11, 12).

Targeting sphingolipids and their effect on inflammation in CVDs has emerged as a promising therapeutic strategy to combat these diseases. Preclinical studies have demonstrated the potential of sphingolipids-based drugs to modulate inflammation and CVDs progression as a consequence, improve endothelial function, and reduce atherosclerosis and cardiovascular events (13).

This review aims to provide an overview of the current understanding of the role of sphingolipids in various CVDs such as atherosclerosis, hypertension, heart failure and stroke, including their involvement in inflammation and lipid metabolism. The review also discusses the potential of sphingolipids-based drugs as a novel therapeutic approach for CVDs, highlighting their advantages and limitations.

## Inflammation and cardiovascular diseases

In recent years, there has been increasing recognition of the role of inflammation, including inflammasome activation, in the development and progression of CVDs. In fact, many studies spanning from the 1990s till now proposed inflammation as a risk factor for CVDs, highlighting the importance of monitoring inflammatory markers such as C-reactive protein (CRP), serum amyloid A (SAA), pro-inflammatory cytokines and CD14++CD16+ monocytes and inflammasomes in predicting cardiovascular events (14–17). These findings have important implications for identifying high-risk individuals and developing targeted interventions to prevent and manage CVDs and are particularly relevant in the context of atherosclerosis, a key underlying process in the development of many CVDs.

Rudolf Virchow, a 19th-century German pathologist, was the first to suggest a link between inflammation and atherosclerosis (18). His discovery of inflammatory cells in atherosclerotic plaques of coronary arteries in histological preparations paved the way for subsequent research into the role of inflammation in this disease. Atherosclerosis is characterized by the accumulation of lipid-rich plaques in the inner lining of arterial walls and is the underlying cause of many CVDs. The inflammatory response in atherosclerosis is initiated by damage or injury to the endothelial cells that line the arterial walls (19, 20). When the endothelium is damaged, it exposes the underlying layers of the arterial wall to circulating blood components, including low-density lipoproteins (LDL). The LDL molecules undergo chemical modifications, such as oxidation, which increase the chance of their uptake by monocytes and macrophages. These immune cells engulf the modified LDL molecules and become foam cells, which accumulate in the arterial wall and contribute to the formation of fatty streaks. The accumulation of foam cells and other immune cells in the arterial wall triggers an inflammatory response (by releasing inflammatory cytokines and chemokines), which perpetuates the cycle of inflammation and plaque formation (21). These immune cells produce enzymes, such as matrix metalloproteinases (MMPs), that break down the extracellular matrix of the arterial wall, leading to the weakening of the wall and making it more prone to rupture (22). The rupture of an atherosclerotic plaque can form a blood clot, which can obstruct blood flow and lead to a heart attack or stroke (23). The production of cytokines and chemokines also contributes to plaque instability and rupture. For example, cytokines such as interleukin-1β (IL-1β) and tumor necrosis factor-alpha (TNF-α) can activate endothelial cells, leading to the expression of adhesion molecules that promote the recruitment of more immune cells to the site of injury (24). In addition, inflammation can also lead to smooth muscle cell proliferation and migration, which can contribute to the thickening of the arterial wall and further narrowing of the blood vessel lumen (25).

The inflammasome, a multiprotein complex that plays a key role in the innate immune response and the regulation of inflammation, was also suggested to play a role in atherosclerosis. It is composed of various proteins, including NLRP3 (Nucleotide-binding domain and Leucine-rich Repeat Protein 3), ASC (Apoptosis-associated Speck-like protein containing a CARD), and pro-caspase-1 (26). Recent mice and human studies have suggested a link between the activation of the inflammasome and the development of cardiovascular diseases. The NLRP3 inflammasome, in particular, has been implicated in promoting inflammation in atherosclerosis (27, 28). Several factors can trigger NLRP3 inflammasome activation, including cholesterol crystals, oxidized lipids, and metabolic dysfunction. Once activated, the inflammasome leads to the production of pro-inflammatory cytokines, such as IL-1β and IL-18, which contribute to the progression of atherosclerosis and other cardiovascular conditions (29).

Furthermore, it was shown that percutaneous coronary interventions (PCI) procedures using stent implantation can increase the already-existent inflammatory response in CAD and cause in-stent restenosis (ISR) and neoatherosclerosis (NA) (30). NA is different from native atherosclerosis and develops due to the combination of chronic inflammation in the vessel wall (which is caused by the stent) with elevated lipoproteins migration to the sub-endothelial space, and can occur months to years following the PCI (31). Therefore, the mechanism of inflammation in atherosclerosis is a complex process involving a variety of immune cells, inflammatory mediators, and signaling pathways.

While atherosclerosis remains the primary cause of numerous cardiovascular diseases, including CAD, heart failure, stroke, and myocardial infarction (MI), not all CVDs are directly associated with atherosclerosis. Extensive research has explored the relationship between inflammation and other CVDs in both human and mice models.

One such example is congenital heart disease (also known as congenital heart defect, CHD). CHD refers to a range of structural and functional abnormalities of the heart that arise from incomplete development of the heart during fetal growth (32). Despite emerging new evidence, the precise role of the immune system and inflammation in CHD remains partially understood. One of these evidence revealed that immune cells such as macrophages may play an imperative role in cardiac development in mice (33).

Furthermore, several human studies examined the relation between CHD and inflammation. Zhang et al. (34) found that individuals with ventricular septal defect had altered levels of three acute phase proteins (proteins whose concentration in the serum change in response to inflammation), implying a possible involvement of inflammation and decreased innate immune system function. Another study indicated that children with structural CHDs have increased levels of pro-inflammatory cytokines TNF-α and IL-6 (35). Finally, Opotowsky et al. (36) demonstrated that adults with CHD with elevated levels of hsCRP (High-sensitivity C-reactive protein), a specific marker of heart disease inflammation, had worse functional status and an increased risk for death or non-elective cardiovascular hospitalization. While recent studies have provided valuable insights into the relationship between inflammation and congenital heart disease, there is still much to learn about the specific pathways involved. Therefore, more research is needed to fully understand these mechanisms and develop targeted interventions that can improve outcomes for patients.

Another condition linked to inflammation is heart arrhythmia. Arrhythmias (or irregular heartbeat) are problems with the rate or rhythm of the heartbeat, triggered by abnormal electric activity which causes the heart to beat irregularly. Atrial fibrillation (AF) is the most common sustained arrhythmia and has a complex pathogenesis. Inflammation has been shown to contribute to the development and progression of AF. Patients with AF showed increased inflammatory markers such as CRP, IL-6, IL-18 and TNF-α (37). Inflammatory processes may promote structural and electrical remodeling of the atria, leading to the initiation and maintenance of AF. Another heart arrhythmia shown to be affected by inflammation is arrhythmogenic cardiomyopathy (AC). AC is a rare genetic disorder characterized by abnormal heart rhythms and progressive damage to the heart muscle, typically caused by mutations in genes that code for proteins in the heart muscle (38). Campian et al. (39) have shown that ARVC (AC that affects the right ventricle) patients had elevated levels of pro-inflammatory cytokines as TNF-α, IL-1β and IL-6 (compared to the control group). The inflammatory response involved T lymphocytes, neutrophils, macrophages, and mast cells (40). Much remains unknown about the role inflammation plays in the development and progression of arrhythmogenic cardiomyopathy and arrhythmias in general. As such, there is a pressing need for further research to untangle the mechanisms involved and identify effective prevention and treatment strategies.

Inflammatory processes are known to be significant contributors to the development and progression of various cardiovascular diseases. While the role of inflammation in atherosclerosis is well-established, its involvement in other cardiovascular conditions, such as arrhythmias and CHD remains an active area of research. Despite significant progress, the complex mechanisms driving these processes are still not fully understood, underscoring the need for further research to identify the specific immune cells, cytokines, and signaling pathways involved. These findings emphasize the importance of addressing chronic inflammation as a possible risk factor for cardiovascular diseases and highlight the potential of novel inflammation-targeted therapies to improve prevention and treatment. Continued research efforts are essential for fully unraveling the complexities of inflammatory processes in cardiovascular diseases.

## Current therapeutic approaches and gaps in cardiovascular diseases

Advancements in the treatment of CVDs have significantly improved patient health and quality of life (41). Despite significant advances in understanding and managing CVDs, there are still considerable gaps in our knowledge and treatment approaches. One of the primary therapeutic approaches for managing CVD is lifestyle changes. These changes include regular exercise, a healthy diet, smoking cessation, weight management, and stress reduction. These lifestyle changes can help prevent the development of CVDs, reduce their risk factors, and improve overall cardiovascular health (42).

In addition, medications are also essential in controlling blood pressure and preventing or treating CVD. Suboptimal control of blood pressure is a major risk factor for CVDs, including cerebrovascular and ischemic heart disease. Research has shown that globally, around 62% of cerebrovascular disease and 49% of ischemic heart disease cases can be attributed to suboptimal control of blood pressure. Therefore, blood pressure-lowering therapy using one or more medications is crucial to CVDs control strategies (43).

Abnormal blood lipids, such as high levels of LDL cholesterol (LDL-C) and low levels of high-density lipoprotein cholesterol (HDL-C), have been established as major risk factors for CVDs. The development of medications to lower lipids, particularly statins, has significantly impacted the prevention and treatment of CVDs. Statins, which are 3-hydroxy-3-methylglutaryl coenzyme A (HMG-CoA) reductase inhibitors, are a class of drugs that have been shown to effectively reduce LDL-C levels and improve lipid profiles (44). In addition, Antiplatelet drugs, such as low-dose aspirin, play a significant role in preventing ischemic heart disease and stroke. These drugs prevent blood clots from forming in the arteries, which can reduce the risk of heart attacks and strokes. The mechanisms of action of major pharmacotherapeutic options for CVDs, including blood pressure-lowering, lipid-lowering, and antiplatelet drugs, work independently from one another. As a result, fixed-dose combinations (FDCs) of these effective medicines have been promoted as a way to simplify treatment and improve adherence (45).

Inflammation is a key process in the development and progression of CVDs, and reducing inflammation may help to prevent or treat these conditions. Therefore, anti-inflammatory drugs have been studied for their potential role in treating and preventing CVDs (46). Several anti-inflammatory drugs have been studied for their effects on CVD, including nonsteroidal anti-inflammatory drugs (NSAIDs), corticosteroids, and monoclonal antibodies that target specific inflammatory pathways (47). NSAIDs, which are commonly used for pain relief, have been associated with an increased risk of cardiovascular events, particularly in individuals with pre-existing CVDs or other risk factors. As a result, the use of NSAIDs in patients with CVDs should be carefully considered and monitored by a healthcare professional. Corticosteroids, such as prednisone, have been shown to reduce inflammation and improve symptoms in patients with various types of CVDs, such as rheumatoid arthritis and vasculitis. However, the long-term use of corticosteroids can have significant side effects, including an increased risk of infections, osteoporosis, and diabetes (47, 48). A combination of standard therapy and canakinumab, a monoclonal antibody that targets IL-1β and the inflammasome, was shown to reduce the risk of heart attack, stroke, or related mortality rates in patients with a history of a heart attack (and have elevated CRP levels) (49) compared to the placebo group. However, it is important to note that canakinumab had a higher incidence of fatal infections. Another anti-inflammatory drug called colchicine (used to treat other conditions such as gout), known for inhibition of IL-1β and migration of leucocytes to inflammation sites (50), was widely studied. It was shown that low-dose colchicine considerably decreased inflammatory markers and the risk of non-cardioembolic ischemic stroke and myocardial infarction (MI) in patients with acute or chronic CAD (51–54). Moreover, emerging evidence from recent researches in mice and humans has linked inflammasomes, which are multiprotein complexes responsible for activating inflammatory responses, to the development and progression of cardiovascular diseases (CVDs), including atherosclerosis. Notably, inflammasomes such as the NOD-like receptor family pyrin domain-containing 3 (NLRP3) inflammasome, have been identified within atherosclerotic plaques, where their activation can contribute to plaque inflammation and instability. Therefore, targeting the inflammasome pathway has emerged as a potential therapeutic strategy for managing CVDs (26).

However, there are still gaps in CVDs management. Many people are unaware of the risk factors for CVDs and how to prevent them. There is a need for greater public awareness campaigns and education initiatives to address this gap (55). Also, there is a need for improved screening and diagnosis of CVDs, particularly in underserved populations who may be at higher risk (56). Another gap is the significant disparities in care access for CVDs, particularly in low-income and rural communities. Improving care access can help reduce the burden of CVDs (57). On the other hand, patients that do get care access do not adhere to their prescribed treatment plan, which can lead to poor health outcomes. There is a need for improved patient education and support to promote adherence (58). The cost of care for CVDs can be prohibitively expensive for many patients, particularly those without insurance or with high out-of-pocket costs. There is a need for more affordable treatment options and improved insurance coverage (59). The use of PCI such as stents, though essential in the management of CVDs, comes with many challenges. The initial bare-metal stents (BMS) can trigger an inflammatory response and lead to ISR and NA (31). Although the new generation of drug-eluting stents coated with slow-release antiproliferative drugs has shown improved outcomes with lower ISR rates and reduced inflammation, studies have indicated a higher incidence of NA (60, 61). These complications highlight the need for more advanced and effective alternatives to optimize patient outcomes.

One of the significant gaps in CVD drug therapy is the effectiveness of statins, which are used to lower cholesterol levels and reduce the risk of cardiovascular events. Although statins have been shown to be effective in reducing the risk of CVDs, there is still a significant proportion of patients who do not respond to statin therapy. Additionally, some patients may experience adverse effects, such as muscle pain or liver damage, which can limit the effectiveness of statins (62, 63). Another significant gap in CVD drug therapy is the effectiveness of antiplatelet agents (APAs), such as aspirin and clopidogrel, in preventing blood clots from forming (64). The safety and efficacy of APAs in patients with chronic kidney disease (CKD) are not well understood due to missing knowledge. One of the major challenges in the use of APAs in CKD patients is the increased risk of major bleeding. CKD patients are at higher risk for bleeding complications, and studies indicate that these patients may also exhibit poor responses to APAs. This may be due to alterations in the metabolism of APAs in CKD patients, as well as other factors such as impaired platelet function and increased inflammation (65). Additionally, there are concerns about the efficacy of APAs in CKD patients. High residual platelet aggregability is linked with higher risk for cardiovascular events, and CKD patients may be at higher risk for residual platelet aggregability despite treatment with APAs (66, 67).

There is no doubt notable progress has been made in treating CVDs, though there is a need for more alternatives, with fewer adverse effects. Future research and innovation are necessary to address the gaps and improve outcomes for individuals affected by CVDs.

## An overview of sphingolipids

**Figure 1:**
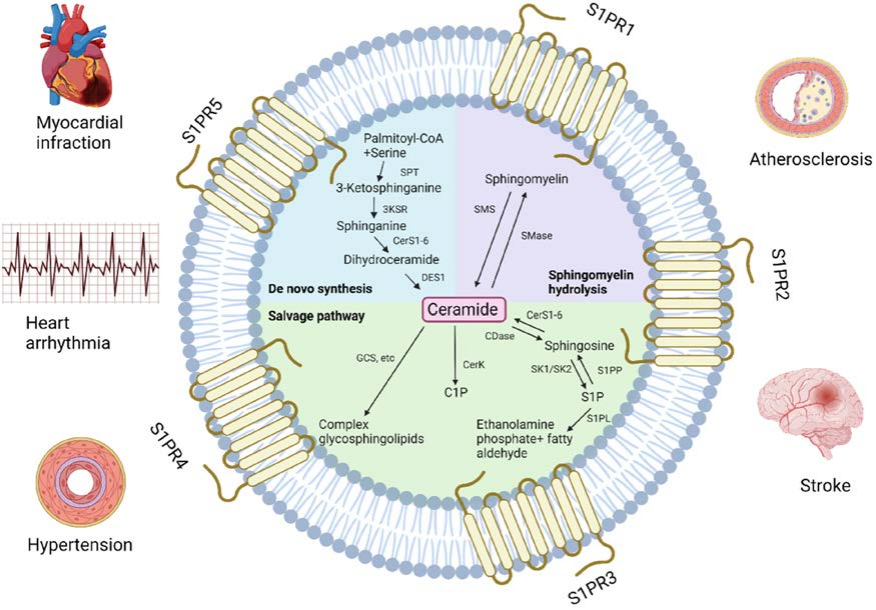
Pathways of sphingolipids metabolism and related cardiovascular diseases. GCS, glucosylceramide synthase; C1P, ceramide-1-phosphate; CerK, ceramide kinase; CerS, ceramide synthase; CDase, ceramidase; SK, sphingosine kinase; S1P, sphingosine-1-phosphate; S1PP, S1P phosphatase; S1PL, S1P lyase; SMase, sphingomyelinase; SMS, sphingomyelin synthase; SPT, serine palmitoyltransferase, 3KSR, 3-ketosphinganine reductase; DES1, dihydroceramide desaturase 1; S1PR, S1P receptor (Created with BioRender.com).

Sphingolipids are structural components of cell membranes essential for cell function in both physiological and therapeutic conditions, managing signaling roles in human health regulation (68). They are a family of complex lipids in all eukaryotes (69) that play essential roles in various cellular processes, including immune response, apoptosis, cell signaling, cell cycle, inflammation, membrane structure, response to stress stimuli, cell adhesion and migration, autophagy, metabolism, nutrient uptake, and cell-cell interactions (70). They comprise 10%–20% of the total lipids in the cell and contain a long-chain amino alcohol called sphingosine, which activates protein kinase C and induces cell cycle arrest apoptosis, attached to a fatty acid by an amide bond (71, 72). They are very similar to phospholipids that contain glycerol instead of sphingosine (73). Ceramides, sphingomyelins, glycosphingolipids, ceramide-1-phosphate (C1P) and sphingosine-1-phosphate (S1P) and are several types of sphingolipids, differentiated by their chemical structure and function (Figure 1).

### Ceramide

Ceramide is often considered a metabolic center because it is critical in sphingolipid formation and catabolism (74). It is also considered the simplest sphingolipid, consisting of a sphingosine backbone and a fatty acid chain. Ceramides are involved in various cellular processes, including inflammation, apoptosis and cell differentiation (75). *De novo* synthesis is one of the three metabolic pathways leading to ceramide formation controlled by ceramide synthase (76). It is generated in the endoplasmic reticulum and then transported to the Golgi (70, 77). Ceramide can be produced in the cell via the hydrolytic pathway in addition to *de novo* biosynthesis. Sphingomyelin is degraded into phosphocholine and ceramide by sphingomyelinase (78). Lastly, in the salvage pathway, ceramide can be synthesized from sphingosine by sphinganine N-acyltransferase (ceramide synthase) (79). Ceramide can be metabolized into complex sphingolipids (sphingomyelins and glycosphingolipids) or converted to C1P (11, 80, 81).

### Sphingomyelin

Sphingomyelin is the most common sphingolipid and a main component of the plasma membrane, consisting of ceramide, a polar phosphorylcholine head group, sphingosine, and a hydrophobic fatty acid chain (82, 83). Sphingomyelin synthesis involves the conversion of serine and palmitoyl-CoA to 3-ketosphinganine, which is reduced to sphinganine, acylated with a fatty acid to form dihydroceramide, desaturated to ceramide, and then converted to sphingomyelin by sphingomyelin synthase (84). Sphingomyelin synthesis mainly occurs in the Golgi apparatus and plasma membrane, and its metabolism creates several products that play major roles in the cells and participate in various signaling pathways (85). The different levels and compositions of sphingomyelin in the cell are regulated by the enzymes of its metabolic pathways, which create a balance between synthesis and degradation. The activity of these enzymes may modify various diseases that eventually bring to the degradation of sphingomyelin and the overproduction of ceramides (84). Sphingomyelin plays a significant role in the formation and maintenance of cell membranes and participates in various signaling pathways. Different fatty acid chains can create unique compositions of binding cholesterol, forming lipid rafts (86). The sphingomyelin level is crucial for cell function (84), especially in nervous tissue and red blood cells, where it contributes to the formation of myelin, acting as an electrical insulator on nerve impulses along the axon.

### Glycosphingolipids

Glycosphingolipids are complex lipids that contain one or more sugar residues attached to the ceramide backbone, in addition to the fatty acid and sphingosine (87). They are produced in the Golgi from glucosylceramide that was transported and glycosylated by specific Golgi-resident enzymes (87–89), located mainly on the outer layer of the cell membrane where the sugar residue is exposed to the cell surface and the ceramide part is inserted in the membrane's external surface (90). Specific enzymes are responsible for the modification and addition of the various sugar molecules resulting in different structures and functions that have an essential role in cell adhesion, molecular signaling, cellular cross-talk (91), and overall membrane organization function (92, 93). However, excessive buildup of glycosphingolipids can cause a range of symptoms such as developmental delay, organ dysfunction, and neurodegeneration (94).

### Sphingosine-1-phosphate and ceramide-1-phosphate

Two other types of sphingolipids are S1P and C1P which are involved in cell proliferation, differentiation, migration, inflammation and survival (95). S1P is produced in the plasma membrane by sphingosine kinase 1 (SphK1) and sphingosine kinase 2 (SphK2) sphingosine kinase isoenzymes by phosphorylation of sphingosine in the cell (96, 97). S1P acts as a signaling molecule by binding to five specific cell surface sphingosine-1-phosphate receptors (S1PR1-5) located on various cell types, including smooth muscle cells, immune cells, and endothelial cells. These S1RP1-5 are involved in various biological processes, such as regulating vascular tone, lymphocyte trafficking, and wound healing (98). In addition to its physiological roles, S1P has been recognized as a probable therapeutic target for several diseases, including cancer, autoimmune disorders, and cardiovascular disease (99–101). C1P is presumed to occur intracellularly through ceramide phosphorylation by CerK (77, 102, 103). Similarly to S1P, C1P functions as a signaling molecule by engaging specific cell surface receptors and is found in immune cells, epithelial cells, and smooth muscle cells (104). These specific signaling receptors regulate inflammation and immunological responses, as well as cell proliferation and survival, and have been discovered as a possible therapeutic target for diseases (105).

Inflammation leads to an increase in the production of S1P and C1P (70). While S1P can be released by activated cells as a normal response, C1P is only released when the membrane is damaged or ruptured. Therefore, C1P is considered a damage-associated molecular pattern since it rapidly and exponentially accumulated at the site of injury (106, 107). Sphingolipids are an essential family of lipids found in all eukaryotes and provide a variety of roles in cellular processes. Ceramide, sphingomyelin, glycosphingolipids, S1P, and C1P are the most frequent forms, distinguished by their chemical structure and function. Each type performs unique and vital roles in various pathways, contributing to cell function and regulation in physiological and therapeutic settings. Understanding sphingolipid metabolism and regulation is critical for treating or preventing CVDs.

## Sphingolipids’ involvement in inflammation and cardiovascular diseases

The involvement of sphingolipids such as ceramide, sphingomyelin, and S1P in the development and progression of cardiovascular diseases is complex and multifaceted and can vary in each disease. Sphingolipids, participate in numerous cellular processes that have the potential to contribute to the development and progression of cardiovascular diseases (108), including inflammation, which is increasingly recognized as a key factor in the pathogenesis of CVDs. In fact, sphingolipids have been shown to play a role in modulating inflammation in different stages of the different CVDs pathophysiology (109). In recent years, an increasing number of studies have shed light on the involvement of sphingolipids in the development of CVDs, as substantiated by an increasing number of studies (108, 110). Recognizing and understanding the relationship between sphingolipids and cardiovascular diseases is significant in effectively managing these diseases and represents an essential step toward developing potential treatments.

There is now substantial evidence, thanks to advances in our understanding of sphingolipid metabolism, animal model studies, and high-level sphingolipidomic techniques, that specific sphingolipid metabolites—like ceramide and S1P—function as signalling molecules that play a significant role in regulating a various cellular process, including immunity, inflammation, and associated disorders (12). Many recent studies have highlighted the role of sphingolipids in regulating inflammation. Phospholipid phosphatase 3 (LPP3), an S1P phosphatase, was shown to promote lymphocyte egress by reducing the levels of S1P in target organs in mice, thus maintaining a blood-tissue S1P gradient (111). Moreover, a specific pool of S1P bound to HDL has been demonstrated to impact on the regulation of lymphopoiesis and neuroinflammation in mice. This is caused by monitoring S1P delivery to the blood and affecting its receptor signalling pathways (110). Studies have suggested a role for SphK1 and S1P in regulating cyclooxygenase 2 (COX2) expression (which generates bioactive prostaglandins involved in inflammation) in rat neonatal cardiac myocytes (112). Ongoing studies suggest that acid sphingomyelinase (SMase), an enzyme involved in ceramide synthesis [which can be secreted or lysosomal (113)], plays a role in sustaining inflammation in intensive care unit patients (after systemic inflammation) by producing of inflammatory cytokines, particularly IL-6 and CC—chemokine ligands (CCL5), in response to TNF-α and IL-1β (114). Levels of serum acid SMase was shown to clinically predict mortality in patients at risk of developing systemic inflammation, potentially due to the induction of acid SMase release by TNF-α (114).

In addition, sphingolipids, such as ceramide and sphingomyelin, have been shown to influence inflammasome formation and activation in the context of CVDs. Ceramide, a key sphingolipid metabolite, can promote inflammasome assembly and activation by inducing mitochondrial dysfunction and the generation of reactive oxygen species (ROS). These processes can trigger the activation of the NOD-like receptor family, pyrin domain-containing 3 (NLRP3) inflammasome pathway. Activation of the NLRP3 inflammasome leads to the maturation and release of pro-inflammatory cytokines, including IL-1β and IL-18, contributing to vascular inflammation and the progression of CVDs (12, 115).

More specifically, extensive research has been made on the involvement of sphingolipids in different CVDs.

### Hypertension

Worldwide, hypertension is responsible for a significant number of premature deaths and cases of cardiovascular disease, accounting for approximately 7.5 million deaths and 57 million disability-adjusted life years (DALYS) (110). The cause-and-effect relationship between sphingolipids and hypertension is not well understood, despite several clinical and experimental studies reporting alterations in sphingolipid metabolism in hypertension diseases (108). The dysregulation of sphingolipid metabolism has been linked to hypertension, with studies demonstrating an increase in total ceramide in SHR and sphingosine levels in hypertension-induced human umbilical vein endothelial cells (HUVECs) (116, 117). Deficiency or inhibition of SphK1 and SphK2, the rate-limiting enzymes in S1P generation, led to decreased blood pressure in mice (118). SphK1 was shown to be upregulated in patients with pulmonary arterial hypertension (PAH) and its genetic deletion and pharmacologic inhibition protected against the development of hypoxia-mediated pulmonary hypertension (HPH) in mice (doi: 10.1164/rccm.201401-0121OC). In addition, SphK1 overexpression in human pulmonary artery smooth muscle cells (PASMCs) resulted in an increase of pro-inflammatory cytokines such as the interleukin family cytokine and the TNF super ligand family (DOI: 10.1007/s12013-021-01006-8) (119). Additionally, elevated levels of circulating S1P have been found to be strongly associated with high blood pressure and inflammation in human and mouse models, making it a powerful biomarker for hypertension (120). Yogi et al. (121) have found that in vascular smooth muscle cells (VSMCs) isolated from rats, S1P induces the activation of pro-inflammatory mediators, and can serve as a biomarker to identify hypertensive rats at high risk of developing cardiovascular diseases. Furthermore, the protein sortilin was found to modulate sphingolipid levels in a way that impaired endothelial function and led to hypertension through an oxidative-stress-dependent mechanism in HUVEC and mice models (and increased the infiltration of inflammatory cells in mesenteric arteries) (117).

The immune system and chronic inflammation have a significant role in the development of hypertension (122). S1P plays a significant role in the regulation of adaptive immune responses as it is crucial for lymphocyte trafficking (123, 124). S1PR1 expression on T cells and the S1P gradient, low in lymphoid organs compared to their exit sites, are required for lymphocyte egress from the thymus and from secondary lymphatic organs to the blood and lymphatic circulation. Deletion of S1PR1 in hematopoietic cells displays disturbed lymphocyte egress from the thymus and secondary lymphoid organs. Meissner et al. (125) showed that inhibition of S1PR1 caused peripheral lymphopenia and prevented the development of hypertension in mice models, thus demonstrating the protentional role of T-cells in hypertension. This study has also demonstrated that SphK2 modulates markers of endothelial activation and inflammation (such as TNF-α, IL-1β and IL-6) in the mesenteric arteries of hypertensive mice.

### Atherosclerosis

Atherosclerosis is an inflammatory condition that can be life-threatening and is characterized by the formation of atheromatous plaques containing cholesterol and other lipids in medium- and large-sized arteries (108). The development of the atherosclerotic lesion is associated with activated macrophages and the presence of various pro-inflammatory cytokines such as TNF-α and IL-1β in mice (126). It has also been shown that macrophages are involved in cholesterol accumulation and plaque formation and promote atherosclerosis by creating foam cells (126). ApoE knockout mice demonstrated that deletion of Akt1/Akt3 is associated with activated macrophages producing pro-inflammatory cytokines leading to the progression of atherosclerotic lesions (127–129). Activated macrophages and pro-inflammatory cytokines can trigger sphingomyelin hydrolysis and ceramide production in conjunction with oxidized low-density lipoprotein (OxLDL) promoting atherogenesis and the formation of atherosclerotic plaques (108).

**Figure 2:**
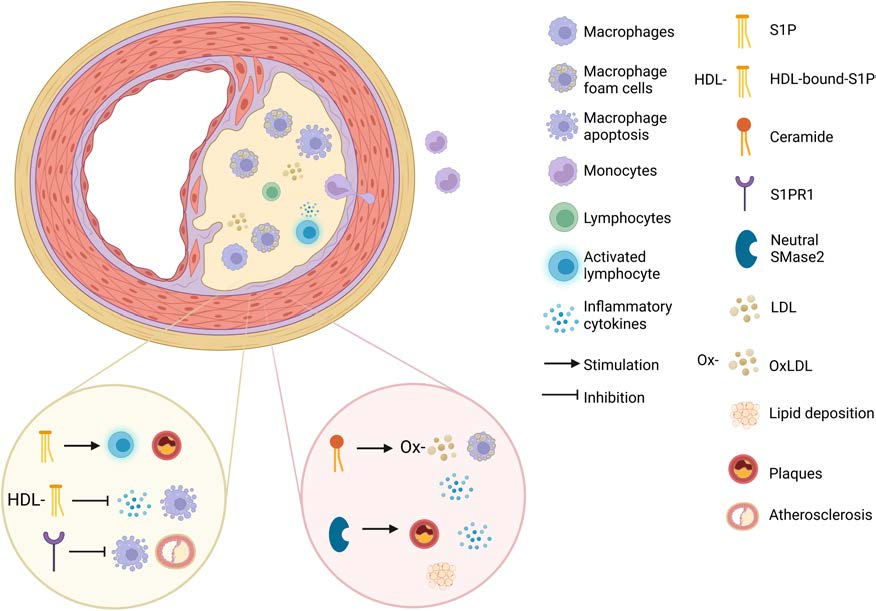
The mechanistic pathways of sphingolipids in atherosclerosis. The effect of S1P (sphingosine-1-phosphate), HDL-bound-S1P, S1PR1 (sphingosine-1-phosphate receptor 1), ceramide and neutral SMase2 (sphingomyelinase 2) on atherosclerosis development (created with BioRender.com).

Interestingly, the role of S1P and its receptors such as S1PR1 in the development of atherosclerosis is multifaceted (Figure 2). Some studies have suggested that S1P can promote atherosclerosis and some indicate that it can mitigate it. S1P can affect lymphocyte circulation, activation and plaque formation contributing to the progression of atherosclerosis in mice, and enhance the expression of adhesion molecules (130). On the contrary some studies have shown S1P suppresses the process of monocyte adhesion (131).

In addition, HDL-bound-S1P was shown to exerts anti-inflammatory effects in HUVECs (132, 133). HDL-bound-S1P was shown to inhibit inducible NO synthase (iNOS) and matrix metalloproteinase 9 (MMP9), both of which promote the inflammatory-related process of atherosclerosis in rat VSMCs (134). In addition, Feuerborn et al. (135). found that HDL-bound-S1P inhibits macrophage apoptosis by activating STAT3 and promoting surviving expression through S1PR2/S1PR3 signaling (in mice and human cell cultures). Additionally, HDL-bound-S1P boosted the formation of the S1PR1-β-arrestin 2 complex and reduced the ability of TNF-α to activate NF-κB and ICAM-1 thus reducing inflammation in HUVECs (133, 134).

Gonzalez et al. (136) have indicated that myeloid-specific S1PR1 deficiency accelerated the development of atherosclerosis as well as the necrotic core formation and the appearance of apoptotic cells within the atherosclerotic plaques of LDL receptor gene (*Ldlr*) deficient mice. On the contrary, S1PR1 selective agonist SEW2871 imparted the protection of macrophages from apoptotic damage through the activation of the PI3K/Akt signaling by endoplasmic reticulum (ER) stress or oxidized LDL. Studies also demonstrated that Increased plasma S1P is helpful in reducing monocyte adhesion and transport across the endothelial layer and minimizing endothelial cell permeability in human cells (137).

Studies have shown that ceramide promotes foam cell formation in mice (138), induces pro-inflammatory cytokine expression in human fibroblasts (139), promotes subendothelial infiltration of OxLDL into the vessel wall in HUVECs (140) And can act as a biomarker for atherosclerosis (Figure 2) (141). Deficiency or inhibition of neutral SMase2 lead to a reduction in atherosclerotic lesions, macrophage infiltration, and lipid deposition, as well as a reduction in IL-1β, IL-6, TNF-α and MCP-1 (monocyte chemoattractant protein-1) in Apolipoprotein E (ApoE)-null mouse models (used as a model of atherosclerosis) (Figure 2) (142). Studies have also found increased plasma concentrations of ceramides, sphingomyelins, sphinganine, and sphingosine in patients with CAD (which is caused by atherosclerosis) (143), and specific ceramide species were associated with increased thrombotic risk, adverse CAD incidents (144), and all-cause mortality, highlighting their potential as biomarkers for improving risk stratification (108, 145).

### Vascular calcification and arterial stiffness

Vascular calcification refers to the deposition of calcium in the walls of blood vessels, particularly arteries. It is a process that occurs as a result of various factors, including aging, chronic inflammation, and certain medical conditions such as atherosclerosis and chronic kidney disease (146). Arterial stiffness, on the other hand, refers to the loss of elasticity or flexibility in the arterial walls, and is also associated with inflammation (147, 148). Arterial stiffness and vascular calcification frequently coincide, implying a strong association between the two. Studies have proposed that vascular calcification may have a contributory role in the development of arterial stiffness (149, 150). Both vascular calcification and arterial stiffness are associated with an increased risk of cardiovascular diseases, including coronary artery disease, stroke and myocardial infraction (151–153). Furthermore, emerging new evidence proposes a connection between sphingolipids and the development of these conditions.

Many studies researched sphingolipids’ involvement in vascular calcification. For instance, transgenic mice overexpressing SphK1 were shown to develop cardiac fibrosis and irregular calcification in the fibrotic area (154). Morris et al. (155) demonstrated that exogenous S1P elevated phosphate-induced VSMCs (isolated from bovine aortic explants) mineralization, whereas C2-ceramide (a ceramide analog) reduced the mineralization. Additionally, the mineralization and S1P levels were decreased by ceramidase and acid SMase inhibition. In the contrary, Luong et al. (156). Have reported that exogenous C2-cermaide elevated the mineralization of human aortic smooth muscle cells (HAOSMCs) which was induced by calcification medium. Bhat et al. (157). Demonstrated that lysosomal Ac (acid ceramidase) deficiency contributes to the development of AMC in the aorta and coronary arteries to smooth muscle-specific acid ceramidase gene knockout mice (Asah1^fl/fl/^SM^Cre^). The same group also revealed that high doses of vitamin D-induced calcification in mice with overexpression of lysosomal acid SMase, resulted in elevated aortic and coronary arterial medial calcification (AMC), and an acid SMase inhibitor decreased this calcification (158). In addition, acid SMase deficiency was shown to inhibit phosphate-induced calcification in culture, mouse model end ex vivo isolated-perfused arteries. Neutral SMase/ceramide pathway was also shown to trigger vascular calcification of human VSMCs (159).

Sphingolipids were also shown to be linked with arterial stiffness. Habibi et al. (160) has demonstrated that while Western diet (WD) increases aortic stiffness in mice models, GW4869, a neutral SMase inhibitor, suppressed the WD-induced increase in neutral SMase activation and pulse wave velocity (PWV), an arterial stiffness marker. Additionally, GW4869 attenuated the WD-induced increased mRNA expression of inflammatory molecules MCP-1, intercellular adhesion molecule 1 (ICAM-1) and vascular CAM-1 (VCAM-1). Several additional sphingolipids were associated with arterial stiffness. Metabolomics profiling among participants of the Bogalusa Heart Study has revealed that metabolites related to sphingomyelin metabolism were correlated with PWV (161). Inhibition of lactosylceramide synthase (LCS) and GCS, two enzymes involved in the synthesis of glycosphingolipids (lactosylceramide and glucosylceramide respectively) improved the PWV of ApoE−/− mice fed a high fat and cholesterol diet (162). Jung et al. (163) has also reported that lactosylceramide served as an independent indicator of elevated arterial stiffness in individuals with impaired fasting glucose.

### Stroke

Stroke is a major cause of mortality globally, and there is a pressing need for effective therapies for both ischemic and hemorrhagic stroke, as well as post-stroke repair. Sphingolipid activities have been found to change after stroke and are closely linked to stroke outcomes, leading to investigations on whether targeting the sphingolipid pathway could be a viable therapeutic approach for stroke (164). Several studies investigated the role of sphingolipids in stroke and cerebral injury. Following ischemic stroke, regulation of S1PR2 antagonist and knockout of S1PR2, which is involved in endothelial activation during acute vascular inflammation injury and increases pro-inflammatory cytokines (165), led to a decrease in infarct ratio and cerebral edema ratio, and better neurological scores in mice cell models of stroke (108, 166). Inhibition of SphK1, a kinase involved in S1P generation and has an essential role in regulating inflammation, also reduced infarct volumes and improved neurological deficits after stroke in mice model by reducing the expression of TRAF2 and NF-κB (108, 167). Mouse brain tissue and human patients with acute ischemic stroke showed a marked increase in long-chain ceramides and specific ceramide species, which correlated with poor functional outcomes (168). The potential use of chloroquine, which usually prevents and treats malaria, for ganglioside dysregulation prevention as a treatment for stroke was also discussed and was associated with decrease in inflammation at the site of injury in stroke-injured rats, but the efficacy of this treatment remains uncertain (108, 169).

### Heart failure

Heart failure is a prevalent disease caused by various factors, including sphingolipids such as ceramides, which contribute to impaired cardiomyocyte function. The accumulation of ceramides in mitochondria and their increased permeability to cytochrome c can lead to apoptosis, a significant mechanism in the development and progression of cardiovascular diseases like heart failure and atherosclerosis (108). Multiple studies have highlighted the significant role that sphingolipids play in the development and progression of this condition. Further understanding of the impact of ceramides on mitochondrial function and apoptosis may lead to the development of more effective treatments for these conditions. Experimental studies in patients with heart failure have shown that there is a decrease in S1P and an increase in ceramide levels in the myocardium, which can directly affect the metabolism and function of the failing heart and inflammatory response (11). Meissner et al. (170) identified the cystic fibrosis transmembrane conductance regulator (CFTR) as a vital control site for S1P signaling in mice models. In heart failure, its TNF-α-dependent reduction underlies an increase in microvascular tone. This research shows that CFTR dysregulation could shift S1P signaling and could represent a unique and important therapeutic target for inflammatory cardiovascular diseases. Dysregulated sphingolipid metabolism genes were observed in human cardiac tissue affected by heart failure, with changes in the gene's expression involved in both the *de novo* and salvage pathways, which are responsible for producing a third of the ceramides in a healthy heart and are activated during inflammation (171, 172). Additionally, an extensive clinical trial involving over 4,000 adults found that higher levels of sphingomyelin and ceramide, which stimulate the production of reactive oxygen species (ROS) in endothelial cells, were associated with an increased risk of heart failure and elevated pro-inflammatory cytokines. These associations were observed independently of other factors. Conversely, higher levels of certain sphingomyelin species were linked to a lower risk of heart failure (108, 173). Inhibition of serine palmitoyltransferase (SPT) with myriocin has shown the ability to reduce adverse cardiac remodeling and improving outcomes in both mice and human studies by inhibiting the initial step of sphingolipids biosynthesis and ceramide accumulation with reduction in very long-chain ceramide species (108, 172). Low-density lipoprotein receptor knockout mice showed elevation in S1P levels that considerably decreased atherosclerosis development due to reduced recruitment of inflammatory activated macrophages and monocytes into the peritoneal cavity, leucocyte adhesion to blood capillary walls and endothelial permeability and plaque formation (174). Furthermore, the severity of heart failure was negatively correlated with plasma S1P levels in patients with ischemic heart disease reducing the inflammatory cytokines and activating reparative markers (175, 176). Additionally, SphK1/S1P/S1PR1 axis was shown to regulate the pro-inflammatory response in mice cardiomyocyte following the induction β1-adrenergic receptor (β1-AR), a chronic inflammatory process caused by myocadiac infraction (177).

Sphingolipids have been found to regulate critical cellular processes, such as inflammation, apoptosis, and oxidative stress, which are all important contributors to the development and progression of cardiovascular diseases. While significant progress has been made in recent years, there are still many unanswered questions that warrant further investigation, such as the role of specific sphingolipid species in different types of cardiovascular diseases. A better understanding of the mechanisms by which sphingolipids contribute to cardiovascular diseases could lead to the development of novel therapeutic strategies for the prevention and treatment of these diseases.

## Sphingolipids-based drugs as a potential therapy for cardiovascular diseases

**Figure 3:**
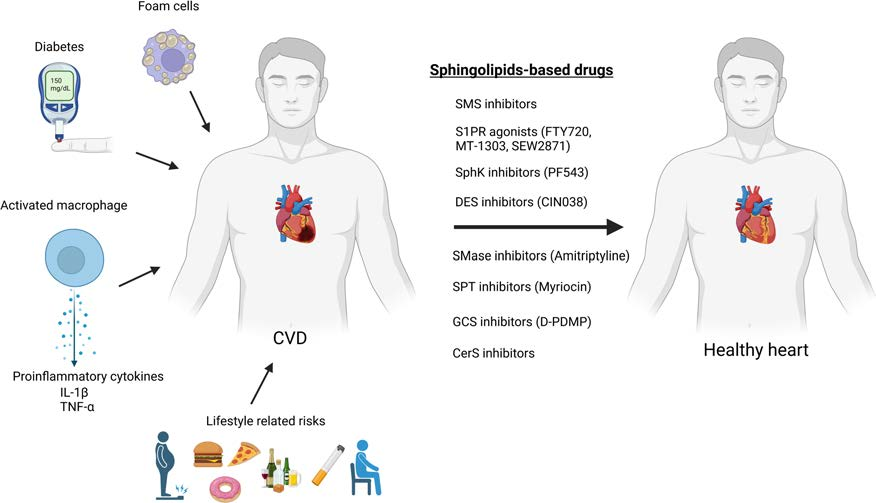
Sphingolipids-based drugs as a protentional therapy for cardiovascular diseases (created with bioRender.com).

To date, there are no drugs for cardiovascular diseases that directly consists of sphingolipids, nonetheless numerous sphingolipid-based drugs (that target sphingolipid metabolism or signaling pathways) have been designed or are presently in development (Table 1; Figure 3). Sphingolipids-based drugs can target the inflammatory process in CVDs or other mechanism involved in the development of CVDs. Most drugs can be divided into groups based on their target such as:

**Table 1:** Drugs targeting sphingolipid metabolism and their effect on CVDs.

Drug	Target	CVDs
Fingolimod	S1PR	Atherosclerosis (178) ↓
		Anti-inflammatory cytokine (178) ↑
		Arrhythmias (179) ↓
		Cardiac hypertrophy (180) ↓
SEW2871	S1PR1	Hypertension (181) ↓
PF543	SphK1	Hypertension (182) ↓
		Pro-inflammatory cytokines (182) ↓
ABC294640	SphK2	Hypertension (125) ↓
Amitriptyline	Acid SMase	Atherosclerosis (183) ↓
Myriocin	SPT	Atherosclerosis (184, 185) ↓
		Plasma lipid levels (184) ↓
D-PDMP	GCS	Atherosclerosis (186) ↓
		Vascular stiffness (186) ↓
CIN038	DES1	Hypertrophic cardiomyopathy (187) ↓
Fenretinide	DES1	Dyslipidemia (188) ↓
		Hypertension (188) ↓
		Pro-inflammatory cytokines (188) ↓
6-[(2R)-4-(4-benzyl-7-chlorophthalazin-1-yl)-2-methylpiperazin-1-yl] pyridine-3-carbonitrile	S1P-lyase	Bradycardia (189) ↓

### S1P and S1PR related inhibitors/agonists

S1PR drugs target the S1PRs that are involved in the regulation of immune cell trafficking and vascular tone. Some S1PR agonists have been shown to be effective in the treatment of multiple sclerosis. They have also been studied for their potential use in the treatment of CVDs such as hypertension and heart failure (190). Fingolimod (FTY720), for instance, indirectly affects sphingolipid metabolism and ceramide synthesis (191). It was shown to prevent ischemia/reperfusion injury-associated arrhythmias in rat heart model (179), inhibit the development of cardiac hypertrophy in stress-induced hypertrophic mice (180), increase anti-inflammatory cytokines in blood concentration and diminish development of atherosclerosis in mice (178). In addition, Imeri et al. (192) showed that fingolimod and its two derivatives (ST-968 and ST-1071) reduce the expression of adhesion molecules of immune cells by activating the S1PR3-PI3K/AKT signaling pathway in human cell lines. However, other studies have shown that multiple sclerosis patients using fingolimod failed to reduce disease progression (193) or have an increased risk of cardiac events (194).

The selective S1PR1 agonist, amiselimod (MT-1303), could pose an alternative since it was shown to have a better cardiac safety profile in a clinical trial (195). Amiselimod binds immune cells inside lymph nodes in multiple sclerosis, stopping them from moving to the brain and spinal cord and creating the inflammation that drives the disease (196). However, additional investigations are required to validate its effectiveness in the treatment of CVDs. SEW2871, an additional S1PR1 selective agonist, reduced systolic blood pressure of hypertensive mice (181). In mice, SEW2817 has been also reported to have anti-inflammatory effects including reducing pro-inflammatory cytokine levels in peritoneal macrophages (197), as well as inhibiting dendritic cell chemotaxis and migration to lymph nodes *in vivo* (198).

In addition, S1P plasma concentrations are related to cardiovascular diseases, including heart rate change, atherosclerosis, heart failure and myocardial infarction. The S1P-lyase inhibitor, 6-[(2R)-4-(4-benzyl-7-chlorophthalazin-1-yl)-2-methylpiperazin-1-yl] pyridine-3-carbonitrile, prevents the degradation of S1P, increase S1P plasma concentrations and slow down the heart rate and consequently improve heart function in rats (189). Furthermore, it was shown to downregulate pro-inflammatory cytokines and reduce inflammatory infiltrates in IBD mice model (199). These targets may prove to be novel treatment avenue for CVDs though further research is needed.

### Sphingosine kinase inhibitors/agonists

These inhibitors are another class of sphingolipid-based drugs that have been studied for their potential use in the treatment of CVDs. Sphingosine kinase is an enzyme that is involved in the synthesis of S1P. Józefczuk et al. (182) have shown that PF543, a selective SphK1 inhibitor, improved the endothelial function of arteries of hypertensive mice. In another study, Wu et al. (200) showed that rats treated with an injection of two different concentrations of PF543 after myocardial infarction, had a reduced expression of the pro-inflammatory cytokines IL-1β, IL-6, and TNF-α. The higher concentration had a better inhibitory effect, and PF543 improved the cardiac function of rats. Shao et al. (201), on the contrary, revealed different results in Oxygen–Glucose Deprivation/Reoxygenation (OGDR)-induced cardiomyocyte injury. K6PC-5 (SphK1 activator) elevated intracellular S1P content in murine cell model and considerably hindered OGDR-induced cardiomyocyte death. Furthermore, SphK1 inhibitors, SKI-II and B-5354c (or SphK1-siRNA knockdown) aggravated OGDR-induced cytotoxicity and even reversed K6PC-5 cytoprotection in rat and murine cell lines. In addition, SKI-II was shown to produce pro-inflammatory cytokines and exacerbate atherosclerosis in low-density lipoprotein receptor-deficient mice on high cholesterol diet (202).

SphK2 antagonists ABC294640 or K-145 considerably decreased the blood pressure levels in Angiotensin-II (AngII)-induced hypertension model in mice (125). Studies have not yet revealed the anti-inflammatory effect of ABC294640 in CVDs. Nonetheless, a previous study in mice ulcerative colitis model, which is associated with an increased risk of CVDs (203), have found that ABC294640 inhibited the activation of NF-κB by TNF-α with some anti-inflammatory effect when administered in a therapeutic perspective with a better gastric safety compared to control NSAID (204). On the contrary, Ishimaru et al. (205) demonstrated that in SphK2-knockout mice, the formation of atherosclerotic lesions was exacerbated. Moreover, SphK2-deficient macrophages had increased lipid content and that could contribute to atherosclerotic plaque formation.

These intriguing and contradicting results regarding SphKs in CVDs require further research to better comprehend SphKs role.

### Acid sphingomyelinase inhibitors

Acid SMase is an enzyme that hydrolyzes sphingomyelin to generate ceramide, and dysregulation of acid SMase activity has been implicated in the development of atherosclerosis and other CVDs (206). Acid SMase inhibitors, such as the compound amitriptyline, have been shown in HUVEC cells to reduce endothelial inflammation and improve vascular endothelial function after preincubation with amitriptyline. The amitriptyline reduced TNF-a-induced Acid SMase/CER and MAPK activation, inhibiting TNF-induced monocyte/EC interactions while decreasing future endothelial inflammation and dysfunction which is linked to atherosclerosis. The preincubation also prevented the downregulation of endothelial nitric oxide synthase (which contributes to atherosclerosis) induced by TNF-α (183). Though, other reports are contradicting regarding the cardioprotective effect of acid SMase inhibition. In 2008, Devlin et al. (207) found that acid SMase deficient mice had reduced lipoprotein retention within early lesions of plaque formation, whereas Leger et al. (184) found that ApoE−/− mice with acid SMase overexpression did not accelerate or exacerbated lesions. Further research is needed to evaluate the efficacy and safety of acid SMase inhibitors in clinical trials.

### Serine palmitoyltransferase inhibitors

SPT catalyzes the condensation of serine and palmitoyl CoA, the first step in the biosynthesis of numerous sphingolipids (184). Myriocin, a commonly used SPT inhibitor, was shown to inhibit atherosclerotic lesions progression, regress pre-existing plaques and lower plasma lipid levels such as ceramides and S1P in ApoE−/− mice (185, 208). In another mice therapeutic study of cystic fibrosis, myriocin was shown to reduce apoptosis and inflammation *in vivo* (209), and in a study conducted by the same group for myocardial reperfusion injury reduction, application of intraventricular administration of nanocarriers-myriocin in mice model during the beginning of reperfusion reduced effectively ceramide accumulation and inflammatory response (210). Though continued study is essential, myriocin and other SPT inhibitors could be potential therapeutic targets for CVDs.

### Glycosphingolipid synthesis inhibitors

Inhibition of different targets in the glycosphingolipid synthesis process has been explored as a possible therapeutic strategy for CVDs. Glucosylceramide synthase (GCS) catalyzes the first reaction of ceramide glycosylation in sphingolipid metabolism (87). Different studies were conducted examining different GSC inhibitors. Chatterjee et al. (162) demonstrated that D-thero-1-phenyl-2-decanoylamino-3-morpholino-1-propanol (D-PDMP), glucosylceramide analog, could mitigate atherosclerosis and vascular stiffness in both ApoE−/− mice. In another atherosclerosis mice study, D-PDMP was found to have anti-inflammatory properties by inhibiting ERK and NF-κB activation and have antioxidative properties as well (211). In addition, Baccam et al. (212) have shown that different GCS inhibitors protect against cardiac hypertrophy in chronic kidney disease in mouse models.

There are many more potential therapeutic targets to treat CVDs that need further investigations. One such example is sphingomyelin synthase (SMS). SMSs catalyze the final step in sphingomyelin biosynthesis. Overexpression of SMS2 (SMS homolog) was shown to promote atherogenesis and elevate plasma sphingomyelin levels in mice models (213). Additionally, inflammatory response and atherosclerotic lesions were reduced in SMS2 deficient mice (214). It's worth noting that the development of highly specific SMS2 inhibitors is crucial for effectively targeting the enzyme and avoiding cross-reactivity with less favorable homologs. The identification of SMS2 selective inhibitors, such as derivatives of 4-benzyloxybenzo[d]isoxazole-3-amine, highlights the therapeutic potential of such compounds, as a specific derivative was shown to reduce chronic inflammation in mice models (215). Ceramide synthases (CerSs) are another example. CerSs are enzymes required for both salvage pathway and *de novo* synthesis of ceramides and other sphingolipids (76). It was shown that CerS5 (a specific CerS) knockdown prevents induction of hypertrophic cardiomyopathy in mice model and inhibits palmitate-induced lipotoxicity in human cardiac progenitor cells (CPCs) (216, 217). In addition, CerS6 inhibition was shown to improve diabetes and obesity indexes in humans (218), thus posing a potential target for cardioprotective approaches. Another potential target is dihydroceramide desaturase (DES), an enzyme that catalyzes the final step in *de novo* ceramide synthesis (219). Heterozygous deletion of the dominant isoform DES1 has been found to avert diet-induced hypertension and vascular dysfunction in mice and reduce the expression of NF-κB in cells isolated from rats (220). Furthermore, CIN038, a selective DES1 inhibitor has been demonstrated to reduce hypertrophy in neonatal rat cardiomyocytes by effecting protein-bound uremic toxins which mediate sphingolipid imbalance and inflammatory responses in heart and kidney cells (187). Fenretinide, another DES1 inhibitor has been found to mitigate the plasma lipid levels mitigate in obese mice thus potentially alleviating diet-induced dyslipidemia (188). In addition, fenretinide has been found to inhibit LPS-induced pro-inflammatory cytokines secretion in mice macrophages cell line and decrease systolic pressure of spontaneously hypertensive rats (SHR) (221). Further studies are needed to better understand DES1's role.

In addition, there are several FDA-approved drugs that are used to treat various conditions and do not directly target sphingolipids metabolism or signaling pathways, while still having cardioprotective properties. Some of these drugs have been shown to reduce ceramide levels, providing a potential therapeutic approach for CVDs. Empagliflozin is a sodium-glucose transport protein 2 (SGLT2) inhibitor used for type 2 diabetes, that was shown to reduce the content of sphingomyelin and ceramide in the heart of type 2 diabetic rats (222). SGLT2 inhibition with empagliflozin, also affects cardiac inflammation by reducing cardiac mRNA levels of IL-6 and TNF-α in rats. In addition, empagliflozin reduces the risk of cardiovascular death or heart failure hospitalization in patients with heart failure, and improves clinical outcomes in these patients (223, 224). Further research is needed to elucidate whether empagliflozin's effect on CVDs is linked to ceramide inhibition.

Although there are currently no CVD drugs directly composed of sphingolipids, several classes of sphingolipid-based drugs are being developed, targeting different pathways involved in sphingolipid metabolism or signaling. These drugs can be grouped based on their targets such as S1PR agonists, sphingosine kinase inhibitors/agonists, acid sphingomyelinase inhibitors, serine palmitoyl transferase inhibitors, glycosphingolipid synthesis inhibitors, and sphingomyelin synthase inhibitors. Various studies indicate the potential of these drugs as therapeutic targets for CVDs, although further research and clinical trials are needed to validate their efficacy and safety.

## Discussion

Though advancements in prevention and treatment have led CVDs global prevalence and mortality to decrease in recent decades (225), they still remain a significant universal health concern. Numerous researchers have investigated the development and progression of CVDs, including inflammation's involvement, in an effort to unravel the complex web of contributors (226, 227). However, as more is uncovered, it becomes increasingly clear that these intricate mechanisms involve a multitude of pathways and factors, and the need for a better alternative and more efficient treatment still remains.

In recent years, sphingolipids have unfolded as a potential therapeutic strategy for CVDs due to their role in the pathogenesis of CVDs (228). The sphingolipid metabolic pathway, which includes multiple enzymes and metabolites such as ceramides, S1P, and sphingomyelin, is a tightly regulated system that has been found to be linked to CVDs (11). Preclinical studies have shown that drugs targeting sphingolipids or their metabolic pathways such as empagliflozin (223, 224), fingolimod (229), and neutral or acid SMases inhibitors (142, 207) have the potential to provide cardioprotective effects by reducing the incidence of cardiovascular events and mortality, as well as modulating inflammation and improving endothelial function.

Despite encouraging preclinical evidence, several challenges and limitations must be addressed before sphingolipid-based drugs can be considered a viable therapeutic alternative for CVDs. Firstly, the pharmacokinetic properties of these drugs need to be optimized to achieve the desired therapeutic effects while minimizing adverse effects. This involves determining the optimal dose, frequency, and route of administration (230).

Secondly, there is a lack of selectivity in targeting sphingolipids, which are a complex class of lipids with multiple functions in cells. Therefore, it is essential to develop sphingolipid-based drugs that can selectively target the desired sphingolipids without affecting others. This requires a better understanding of the specific sphingolipid pathways involved in CVDs and the development of drugs that can selectively target these pathways (231). Specific targeting strategies, such as the use of targeted nanoparticles, can help reduce off-target effects and enhance the efficacy of sphingolipid-based drugs (232).

Lastly, more rigorous clinical trials are needed to evaluate the efficacy and safety of sphingolipid-based drugs. Most current clinical trials evaluating the efficacy of these drugs are small-scale and have conflicting results. Therefore, there is a need for larger, well-designed clinical trials to establish their efficacy and safety in the treatment of CVDs (233). Furthermore, most preclinical studies on sphingolipid-based drugs focused on their short-term effects, and their long-term effects are not well understood. Thus, more extensive preclinical studies are needed to establish their long-term safety (234).

In conclusion, while there are still challenges to be addressed in the development and optimization of sphingolipid-based drugs for the treatment of cardiovascular diseases, the promising results of ongoing research suggest that with continued efforts to design safe and effective drugs, optimize pharmacokinetics, and enhance specificity in targeting sphingolipids, sphingolipid-based drugs could become a viable therapeutic option for CVDs in the future.

## References

[1] Dal Canto E Ceriello A Rydén L Ferrini M Hansen TB Schnell O Diabetes as a cardiovascular risk factor: an overview of global trends of macro and micro vascular complications. Eur J Prev Cardiol. (2019) 26(2 Suppl):25–32. 10.1177/2047487319878371 31722562

[2] World Health Organization. Cardiovascular diseases (CVDs). World Health Organization (2021).

[3] Amen OM Sarker SD Ghildyal R Arya A. Endoplasmic reticulum stress activates unfolded protein response signaling and mediates inflammation, obesity, and cardiac dysfunction: therapeutic and molecular approach. Front Pharmacol. (2019) 10:472324. 10.3389/fphar.2019.00977 PMC6747043 31551782

[4] Sánchez-Gloria JL Arellano-Buendía AS Juárez-Rojas JG García-Arroyo FE Argüello-García R Sánchez-Muñoz F Cellular mechanisms underlying the cardioprotective role of allicin on cardiovascular diseases. Int J Mol Sci. (2022) 23(16):9082. 10.3390/ijms23169082 36012349 PMC9409331

[5] Libby P. Inflammation and cardiovascular disease mechanisms. Am J Clin Nutr. (2006) 83(2):456S–60S. 10.1093/ajcn/83.2.456S 16470012

[6] Chen L Deng H Cui H Fang J Zuo Z Deng J Inflammatory responses and inflammation-associated diseases in organs. Oncotarget. (2018) 9(6):7204–18. 10.18632/oncotarget.23208 29467962 PMC5805548

[7] Wasserman AH Venkatesan M Aguirre A. Bioactive lipid signaling in cardiovascular disease, development, and regeneration. Cells. (2020) 9(6):1391. 10.3390/cells9061391 32503253 PMC7349721

[8] Mattisson IY Christoffersen C. Apolipoprotein M and its impact on endothelial dysfunction and inflammation in the cardiovascular system. Atherosclerosis. (2021) 334:76–84. 10.1016/j.atherosclerosis.2021.08.039 34482091

[9] Li A Gao M Liu B Qin Y Chen L Liu H Mitochondrial autophagy: molecular mechanisms and implications for cardiovascular disease. Cell Death Dis. (2022) 13(5):444. 10.1038/s41419-022-04906-6 35534453 PMC9085840

[10] Alfaddagh A Martin SS Leucker TM Michos ED Blaha MJ Lowenstein CJ Inflammation and cardiovascular disease: from mechanisms to therapeutics. Am J Prev Cardiol. (2020) 4:100130. 10.1016/j.ajpc.2020.100130 34327481 PMC8315628

[11] Borodzicz-Jażdżyk S Jażdżyk P Łysik W Cudnoch-Jȩdrzejewska A Czarzasta K. Sphingolipid metabolism and signaling in cardiovascular diseases. Front Cardiovasc Med. (2022) 9:915961. 10.3389/fcvm.2022.915961 36119733 PMC9471951

[12] Maceyka M Spiegel S. Sphingolipid metabolites in inflammatory disease. Nature. (2014) 510(7503):58–67. 10.1038/nature13475 24899305 PMC4320971

[13] Green CD Maceyka M Cowart LA Spiegel S. Sphingolipids in metabolic disease: the good, the bad, and the unknown. Cell Metab. (2021) 33(7):1293–306. 10.1016/j.cmet.2021.06.006 34233172 PMC8269961

[14] Rogacev KS Cremers B Zawada AM Seiler S Binder N Ege P CD14++CD16+ monocytes independently predict cardiovascular events. J Am Coll Cardiol. (2012) 60(16):1512–20. 10.1016/j.jacc.2012.07.019 22999728

[15] Kaptoge S Seshasai SRK Gao P Freitag DF Butterworth AS Borglykke A Inflammatory cytokines and risk of coronary heart disease: new prospective study and updated meta-analysis. Eur Heart J. (2014) 35(9):578–89. 10.1093/eurheartj/eht367 24026779 PMC3938862

[16] Ridker PM Cushman M Stampfer MJ Tracy RP Hennekens CH. Inflammation, aspirin, and the risk of cardiovascular disease in apparently healthy men. N Engl J Med. (1997) 336(14):973–9. 10.1056/NEJM199704033361401 9077376

[17] Liuzzo G Biasucci LM Gallimore JR Grillo RL Rebuzzi AG Pepys MB The prognostic value of C-reactive protein and Serum amyloid A protein in severe unstable angina. N Engl J Med. (1994) 331(7):417–24. 10.1056/NEJM199408183310701 7880233

[18] Virchow R. As based upon physiological and pathological histology. Nutr Rev. (2009) 47(1):23–5. 10.1111/j.1753-4887.1989.tb02747.x 2649802

[19] Libby P Ridker PM Hansson GK. Inflammation in atherosclerosis. J Am Coll Cardiol. (2009) 54(23):2129–38. 10.1016/j.jacc.2009.09.009 19942084 PMC2834169

[20] Ross R. Atherosclerosis—an inflammatory disease. N Engl J Med. (1999) 340(2):115–26. 10.1056/NEJM199901143400207 9887164

[21] Raggi P Genest J Giles JT Rayner KJ Dwivedi G Beanlands RS Role of inflammation in the pathogenesis of atherosclerosis and therapeutic interventions. Atherosclerosis. (2018) 276:98–108. 10.1016/j.atherosclerosis.2018.07.014 30055326

[22] Bobryshev YV Ivanova EA Chistiakov DA Nikiforov NG Orekhov AN. Macrophages and their role in atherosclerosis: pathophysiology and transcriptome analysis. Biomed Res Int. (2016) 2016:1–13. 10.1155/2016/9582430 PMC4967433 27493969

[23] Lusis AJ. Atherosclerosis. Nature. (2000) 407(6801):233–41. 10.1038/35025203 11001066 PMC2826222

[24] Zhang C. The role of inflammatory cytokines in endothelial dysfunction. Basic Res Cardiol. (2008) 103(5):398–406. 10.1007/s00395-008-0733-0 18600364 PMC2705866

[25] Jaminon A Reesink K Kroon A Schurgers L. The role of vascular smooth muscle cells in arterial remodeling: focus on calcification-related processes. Int J Mol Sci. (2019) 20(22):5694. 10.3390/ijms20225694 31739395 PMC6888164

[26] Olsen MB Gregersen I Sandanger Ø Yang K Sokolova M Halvorsen BE Targeting the inflammasome in cardiovascular disease. JACC Basic Transl Sci. (2022) 7(1):84–98. 10.1016/j.jacbts.2021.08.006 35128212 PMC8807732

[27] Liao Y Liu K Zhu L. Emerging roles of inflammasomes in cardiovascular diseases. Front Immunol. (2022) 13:834289. 10.3389/fimmu.2022.834289 35464402 PMC9021369

[28] Garg NJ. Inflammasomes in cardiovascular diseases. Am J Cardiovasc Dis. (2011) 1(3):244–54. 22254202 PMC3253520

[29] Jin Y Fu J. Novel insights into the NLRP3 inflammasome in atherosclerosis. J Am Heart Assoc. (2019) 8(12):834289. 10.1161/JAHA.119.012219 PMC6645652 31184236

[30] Hetherington I Totary-Jain H. Anti-atherosclerotic therapies: milestones, challenges, and emerging innovations. Mol Ther. (2022) 30(10):3106–17. 10.1016/j.ymthe.2022.08.024 36065464 PMC9552812

[31] Ochijewicz D Tomaniak M Opolski G Kochman J. Inflammation as a determinant of healing response after coronary stent implantation. Int J Cardiovasc Imaging. (2021) 37(3):791–801. 10.1007/s10554-020-02073-3 33479786 PMC7969567

[32] Centers for Disease Control and Prevention. Congenital heart defects (CHDs) (2023). Available at: https://www.cdc.gov/ncbddd/heartdefects/facts.html (Accessed May 1, 2023).

[33] Swirski FK Nahrendorf M. Cardioimmunology: the immune system in cardiac homeostasis and disease. Nat Rev Immunol. (2018) 18(12):733–44. 10.1038/s41577-018-0065-8 30228378

[34] Zhang X Wang K Yang Q Wang J Xuan C Liu XC Acute phase proteins altered in the plasma of patients with congenital ventricular septal defect. Proteomics Clin Appl. (2015) 9(11–12):1087–96. 10.1002/prca.201400166 25914298

[35] Sharma R Bolger AP Li W Davlouros PA Volk HD Poole-Wilson PA Elevated circulating levels of inflammatory cytokines and bacterial endotoxin in adults with congenital heart disease. Am J Cardiol. (2003) 92(2):188–93. 10.1016/S0002-9149(03)00536-8 12860222

[36] Opotowsky AR Valente AM Alshawabkeh L Cheng S Bradley A Rimm EB Prospective cohort study of C-reactive protein as a predictor of clinical events in adults with congenital heart disease: results of the Boston adult congenital heart disease biobank. Eur Heart J. (2018) 39(34):3253–61. 10.1093/eurheartj/ehy362 30010900 PMC6127895

[37] Luan Y Guo Y Li S Yu B Zhu S Li S Interleukin-18 among atrial fibrillation patients in the absence of structural heart disease. Europace. (2010) 12(12):1713–8. 10.1093/europace/euq321 20833691

[38] Shah SN Umapathi KK Oliver TI. Arrhythmogenic right ventricular cardiomyopathy. StatPearls (2022). Available at: https://www.ncbi.nlm.nih.gov/books/NBK470378/#_NBK470378_pubdet_ (Accessed May 1, 2023) 29262224

[39] Campian ME Verberne HJ Hardziyenka M de Groot EAA van Moerkerken AF van Eck-Smit BLF Assessment of inflammation in patients with arrhythmogenic right ventricular cardiomyopathy/dysplasia. Eur J Nucl Med Mol Imaging. (2010) 37(11):2079–85. 10.1007/s00259-010-1525-y 20603720 PMC2948173

[40] Campuzano O Alcalde M Iglesias A Barahona-Dussault C Sarquella-Brugada G Benito B Arrhythmogenic right ventricular cardiomyopathy: severe structural alterations are associated with inflammation. J Clin Pathol. (2012) 65(12):1077–83. 10.1136/jclinpath-2012-201022 22944624

[41] Kandaswamy E Zuo L. Recent advances in treatment of coronary artery disease: role of science and technology. Int J Mol Sci. (2018) 19(2):424. 10.3390/ijms19020424 29385089 PMC5855646

[42] Masana L Ros E Sudano I Angoulvant D Ibarretxe Gerediaga D Murga Eizagaechevarria N Is there a role for lifestyle changes in cardiovascular prevention? What, when and how? Atheroscler Suppl. (2017) 26:2–15. 10.1016/S1567-5688(17)30020-X 28434481

[43] De Geest S Sabaté E. Adherence to long-term therapies: evidence for action. Eur J Cardiovasc Nurs. (2003) 2(4):323–323. 10.1016/S1474-5151(03)00091-4 14667488

[44] Jamison DT Breman JG Measham AR Alleyne G Claeson M Evans DB Disease control priorities in developing countries. 2nd edition. Washington (DC): The International Bank for Reconstruction and Development / The World Bank (2006). Available from: https://www.ncbi.nlm.nih.gov/books/NBK11728/ Co-published by Oxford University Press, New York 21250309

[45] World Health Organization. Priority medicines for Europe and the world / Warren Kaplan, Richard Laing. World Health Organization (2004). https://apps.who.int/iris/handle/10665/68769

[46] Golia E Limongelli G Natale F Fimiani F Maddaloni V Pariggiano I Inflammation and cardiovascular disease: from pathogenesis to therapeutic target. Curr Atheroscler Rep. (2014) 16(9):435. 10.1007/s11883-014-0435-z 25037581

[47] Ghlichloo I Gerriets V. Nonsteroidal anti-inflammatory drugs (NSAIDs). In: Treasure Island (FL): StatPearls. StatPearls Publishing (2022). PMID: . Available at: https://europepmc.org/article/nbk/nbk547742 (Accessed May 1, 2023). 31613522

[48] Schjerning AM McGettigan P Gislason G. Cardiovascular effects and safety of (non-aspirin) NSAIDs. Nat Rev Cardiol. (2020) 17(9):574–84. 10.1038/s41569-020-0366-z 32322101

[49] Ridker PM Everett BM Thuren T MacFadyen JG Chang WH Ballantyne C Antiinflammatory therapy with canakinumab for atherosclerotic disease. N Engl J Med. (2017) 377(12):1119–31. 10.1056/NEJMoa1707914 28845751

[50] D’Amario D Cappetta D Cappannoli L Princi G Migliaro S Diana G Colchicine in ischemic heart disease: the good, the bad and the ugly. Clin Res Cardiol. (2021) 110(10):1531–42. 10.1007/s00392-021-01828-9 33713178 PMC8484100

[51] Nidorf SM Eikelboom JW Budgeon CA Thompson PL. Low-dose colchicine for secondary prevention of cardiovascular disease. J Am Coll Cardiol. (2013) 61(4):404–10. 10.1016/j.jacc.2012.10.027 23265346

[52] Nidorf SM Fiolet ATL Mosterd A Eikelboom JW Schut A Opstal TSJ Colchicine in patients with chronic coronary disease. N Engl J Med. (2020) 383(19):1838–47. 10.1056/NEJMoa2021372 32865380

[53] Tardif JC Kouz S Waters DD Bertrand OF Diaz R Maggioni AP Efficacy and safety of low-dose colchicine after myocardial infarction. N Engl J Med. (2019) 381(26):2497–505. 10.1056/NEJMoa1912388 31733140

[54] Bouabdallaoui N Tardif JC Waters DD Pinto FJ Maggioni AP Diaz R Time-to-treatment initiation of colchicine and cardiovascular outcomes after myocardial infarction in the colchicine cardiovascular outcomes trial (COLCOT). Eur Heart J. (2020) 41(42):4092–9. 10.1093/eurheartj/ehaa659 32860034 PMC7700755

[55] Ostrowski-Winkler LA, The effects of education on cardiovascular disease knowledge. Evidence-Based practice project reports (2014).

[56] Opoku-Acheampong AA Rosenkranz RR Adhikari K Muturi N Logan C Kidd T. Tools for assessing cardiovascular disease risk factors in underserved young adult populations: a systematic review. Int J Environ Res Public Health. (2021) 18(24):13305. 10.3390/ijerph182413305 34948914 PMC8707965

[57] Artiga S Orgera K Pham O. Disparities in health and health care: Five key questions and answers. San Francisco, CA: Kaiser Family Foundation (2020).

[58] Xu HY Yu YJ Zhang QH Hu HY Li M. Tailored interventions to improve medication adherence for cardiovascular diseases. Front Pharmacol. (2020) 11:510339. 10.3389/fphar.2020.510339 33364935 PMC7751638

[59] Gaziano TA Suhrcke M Brouwer E Levin C Nikolic I Nugent R. Costs and cost-effectiveness of interventions and policies to prevent and treat cardiovascular and respiratory diseases. In: Prabhakaran D, Anand S, Gaziano TA, et al. editors. Cardiovascular, Respiratory, and Related Disorders. 3rd edition. Washington (DC): The International Bank for Reconstruction and Development / The World Bank; Chapter 19 (2017). Available from: https://www.ncbi.nlm.nih.gov/books/NBK525142/ 10.1596/978-1-4648-0518-9_ch19 30212069

[60] Romero ME Yahagi K Kolodgie FD Virmani R. Neoatherosclerosis from a pathologist’s point of view. Arterioscler Thromb Vasc Biol. (2015) 35(10):e43–e9. 10.1161/ATVBAHA.115.306251 26399921

[61] Nusca A Viscusi MM Piccirillo F De Filippis A Nenna A Spadaccio C In stent neo-atherosclerosis: pathophysiology, clinical implications, prevention, and therapeutic approaches. Life. (2022) 12(3):393. 10.3390/life12030393 35330144 PMC8955389

[62] Yourman LC Cenzer IS Boscardin WJ Nguyen BT Smith AK Schonberg MA Evaluation of time to benefit of statins for the primary prevention of cardiovascular events in adults aged 50 to 75 years. JAMA Intern Med. (2021) 181(2):179. 10.1001/jamainternmed.2020.6084 33196766 PMC7670393

[63] Ramkumar S Raghunath A Raghunath S. Statin therapy: review of safety and potential side effects. Acta Cardiol Sin. (2016) 32(6):631–9. 10.6515/ACS20160611A 27899849 PMC5126440

[64] Santos BC Flumignan RL Civile VT Atallah ÁN Nakano LC. Prophylactic anticoagulants for non-hospitalised people with COVID-19. Cochrane Database Syst Rev. (2022) 2022(4). 10.1002/14651858.CD015102 PMC10428666 37591523

[65] Baigent C Landray M Leaper C Altmann P Armitage J Baxter A First United Kingdom heart and renal protection (UK-HARP-I) study: biochemical efficacy and safety of simvastatin and safety of low-dose aspirin in chronic kidney disease. Am J Kidney Dis. (2005) 45(3):473–84. 10.1053/j.ajkd.2004.11.015 15754269

[66] Jain N Hedayati SS Sarode R Banerjee S Reilly RF. Antiplatelet therapy in the management of cardiovascular disease in patients with CKD. Clin J Am Soc Nephrol. (2013) 8(4):665–74. 10.2215/CJN.06790712 23024160 PMC5972379

[67] Morel O El Ghannudi S Jesel L Radulescu B Meyer N Wiesel ML Cardiovascular mortality in chronic kidney disease patients undergoing percutaneous coronary intervention is mainly related to impaired P2Y12 inhibition by clopidogrel. J Am Coll Cardiol. (2011) 57(4):399–408. 10.1016/j.jacc.2010.09.032 21251579

[68] Yang F Chen G. The nutritional functions of dietary sphingomyelin and its applications in food. Front Nutr. (2022) 9:1002574. 10.3389/fnut.2022.1002574 36337644 PMC9626766

[69] Breslow DK. Sphingolipid homeostasis in the endoplasmic Reticulum and beyond. Cold Spring Harb Perspect Biol. (2013) 5(4):a013326. 10.1101/cshperspect.a013326 23545423 PMC3683901

[70] Hannun YA Obeid LM. Sphingolipids and their metabolism in physiology and disease. Nat Rev Mol Cell Biol. (2018) 19(3):175–91. 10.1038/nrm.2017.107 29165427 PMC5902181

[71] Chalfant CE Spiegel S. Sphingosine 1-phosphate and ceramide 1-phosphate: expanding roles in cell signaling. J Cell Sci. (2005) 118(20):4605–12. 10.1242/jcs.02637 16219683

[72] Bennett MK Wallington-Beddoe CT Pitson SM. Sphingolipids and the unfolded protein response. Biochim Biophys Acta Mol Cell Biol Lipids. (2019) 1864(10):1483–94. 10.1016/j.bbalip.2019.06.002 31176037

[73] Iessi E Marconi M Manganelli V Sorice M Malorni W Garofalo T On the role of sphingolipids in cell survival and death. Int Rev Cell Mol Biol. (2020) 351:149–95. 10.1016/bs.ircmb.2020.02.004 32247579

[74] Hannun YA Obeid LM. Principles of bioactive lipid signalling: lessons from sphingolipids. Nat Rev Mol Cell Biol. (2008) 9(2):139–50. 10.1038/nrm2329 18216770

[75] Burgert A Schlegel J Bécam J Doose S Bieberich E Schubert-Unkmeir A Characterization of plasma membrane ceramides by super-resolution microscopy. Angew Chem Int Ed. 2017;56(22):6131–5. 10.1002/anie.201700570 PMC5549273 28379629

[76] Mullen TD Hannun YA Obeid LM. Ceramide synthases at the centre of sphingolipid metabolism and biology. Biochem J. (2012) 441(3):789–802. 10.1042/BJ20111626 22248339 PMC3689921

[77] Shinghal R Scheller RH Bajjalieh SM. Ceramide 1-phosphate phosphatase activity in brain. J Neurochem. (1993) 61(6):2279–85. 10.1111/j.1471-4159.1993.tb07470.x 8245978

[78] Bartke N Hannun YA. Bioactive sphingolipids: metabolism and function. J Lipid Res. (2009) 50:S91–6. 10.1194/jlr.R800080-JLR200 19017611 PMC2674734

[79] Borodzicz S Czarzasta K Kuch M Cudnoch-Jedrzejewska A. Sphingolipids in cardiovascular diseases and metabolic disorders. Lipids Health Dis. (2015) 14(1):55. 10.1186/s12944-015-0053-y 26076974 PMC4470334

[80] Gault CR Obeid LM Hannun YA. An overview of sphingolipid metabolism: from synthesis to breakdown. Adv Exp Med Biol (2010) 688:1–23. 10.1007/978-1-4419-6741-1_1 20919643 PMC3069696

[81] Kihara A. Synthesis and degradation pathways, functions, and pathology of ceramides and epidermal acylceramides. Prog Lipid Res. (2016) 63:50–69. 10.1016/j.plipres.2016.04.001 27107674

[82] Stillwell W. An introduction to biological membranes: from bilayers to rafts. In: Stillwell W, editor. Newnes. Elsevier (2013).

[83] Filippov A Orädd G Lindblom G. Sphingomyelin structure influences the lateral diffusion and raft formation in lipid bilayers. Biophys J. (2006) 90(6):2086–92. 10.1529/biophysj.105.075150 16387761 PMC1386786

[84] Bienias K Fiedorowicz A Sadowska A Prokopiuk S Car H. Regulation of sphingomyelin metabolism. Pharmacol Rep. (2016) 68(3):570–81. 10.1016/j.pharep.2015.12.008 26940196

[85] Ramstedt B Slotte JP. Membrane properties of sphingomyelins. FEBS Lett. (2002) 531(1):33–7. 10.1016/S0014-5793(02)03406-3 12401199

[86] Needham D Nunn RS. Elastic deformation and failure of lipid bilayer membranes containing cholesterol. Biophys J. (1990) 58(4):997–1009. 10.1016/S0006-3495(90)82444-9 2249000 PMC1281045

[87] D’Angelo G Capasso S Sticco L Russo D. Glycosphingolipids: synthesis and functions. FEBS J. (2013) 280(24):6338–53. 10.1111/febs.12559 24165035

[88] van Meer G Voelker DR Feigenson GW. Membrane lipids: where they are and how they behave. Nat Rev Mol Cell Biol. (2008) 9(2):112–24. 10.1038/nrm2330 18216768 PMC2642958

[89] Buton X Hervé P Kubelt J Tannert A Burger KNJ Fellmann P Transbilayer movement of monohexosylsphingolipids in endoplasmic Reticulum and Golgi membranes. Biochemistry. (2002) 41(43):13106–15. 10.1021/bi020385t 12390039

[90] Tettamanti G. Ganglioside/glycosphingolipid turnover: new concepts. Glycoconj J. (2003) 20(5):301–17. 10.1023/B:GLYC.0000033627.02765.cc 15229395

[91] Sproul TW Malapati S Kim J Pierce SK. Cutting edge: b cell antigen receptor signaling occurs outside lipid rafts in immature B cells. J Immunol. (2000) 165(11):6020–3. 10.4049/jimmunol.165.11.6020 11086032

[92] Holthuis JCM Pomorski T Raggers RJ Sprong H Van Meer G. The organizing potential of sphingolipids in intracellular membrane transport. Physiol Rev. (2001) 81(4):1689–723. 10.1152/physrev.2001.81.4.1689 11581500

[93] Hakomori S-I. Structure and function of glycosphingolipids and sphingolipids: recollections and future trends. Biochim Biophys Acta. (2008);1780(3):325–46. 10.1016/j.bbagen.2007.08.015 17976918 PMC2312460

[94] Platt FM d’Azzo A Davidson BL Neufeld EF Tifft CJ. Lysosomal storage diseases. Nat Rev Dis Primers. (2018) 4(1):27. 10.1038/s41572-018-0025-4 30275469

[95] Proia RL Hla T. Emerging biology of sphingosine-1-phosphate: its role in pathogenesis and therapy. J Clin Invest. (2015) 125(4):1379–87. 10.1172/JCI76369 25831442 PMC4409021

[96] Maceyka M Harikumar KB Milstien S Spiegel S. Sphingosine-1-phosphate signaling and its role in disease. Trends Cell Biol. (2012) 22(1):50–60. 10.1016/j.tcb.2011.09.003 22001186 PMC3253987

[97] Weigert A Olesch C Brüne B. Sphingosine-1-phosphate and macrophage biology—how the Sphinx tames the big eater. Front Immunol. (2019) 10:469023. 10.3389/fimmu.2019.01706 PMC6658986 31379883

[98] Kleuser B Bäumer W. Sphingosine 1-phosphate as essential signaling molecule in inflammatory skin diseases. Int J Mol Sci. (2023) 24(2):1456. 10.3390/ijms24021456 36674974 PMC9863039

[99] Forrest M Sun SY Hajdu R Bergstrom J Card D Doherty G Immune cell regulation and cardiovascular effects of sphingosine 1-phosphate receptor agonists in rodents are mediated via distinct receptor subtypes. J Pharmacol Exp Ther. (2004) 309(2):758–68. 10.1124/jpet.103.062828 14747617

[100] Scott FL Clemons B Brooks J Brahmachary E Powell R Dedman H Ozanimod (RPC1063) is a potent sphingosine-1-phosphate receptor-1 (S1P 1) and receptor-5 (S1P 5) agonist with autoimmune disease-modifying activity. Br J Pharmacol. (2016);173(11):1778–92. 10.1111/bph.13476 26990079 PMC4867749

[101] Wang P Yuan Y Lin W Zhong H Xu K Qi X. Roles of sphingosine-1-phosphate signaling in cancer. Cancer Cell Int. (2019) 19(1):295. 10.1186/s12935-019-1014-8 31807117 PMC6857321

[102] Ichikawa S Hirabayashi Y. Glucosylceramide synthase and glycosphingolipid synthesis. Trends Cell Biol. (1998) 8(5):198–202. 10.1016/S0962-8924(98)01249-5 9695839

[103] Presa N Gomez-Larrauri A Rivera IG Ordoñez M Trueba M Gomez-Muñoz A. Regulation of cell migration and inflammation by ceramide 1-phosphate. Biochim Biophys Acta. (2016);1861(5):402–9. 10.1016/j.bbalip.2016.02.007 26875839

[104] Presa N Gomez-Larrauri A Dominguez-Herrera A Trueba M Gomez-Muñoz A. Novel signaling aspects of ceramide 1-phosphate. Biochim Biophys Acta Mol Cell Biol Lipids. (2020);1865(4):158630. 10.1016/j.bbalip.2020.158630 31958571

[105] Mena HA Zubiry PR Dizier B Mignon V Parborell F Schattner M Ceramide 1-phosphate protects endothelial colony–forming cells from apoptosis and increases vasculogenesis in vitro and in vivo. Arterioscler Thromb Vasc Biol. (2019) 39(10):e219–e32. 10.1161/ATVBAHA.119.312766 31434496

[106] Tan-Chen S Guitton J Bourron O Le Stunff H Hajduch E. Sphingolipid metabolism and signaling in skeletal muscle: from physiology to physiopathology. Front Endocrinol (Lausanne). (2020) 11:557432. 10.3389/fendo.2020.00491 PMC7426366 32849282

[107] Mishra SK Gao YG Zou X Stephenson DJ Malinina L Hinchcliffe EH Emerging roles for human glycolipid transfer protein superfamily members in the regulation of autophagy, inflammation, and cell death. Prog Lipid Res. (2020) 78:101031. 10.1016/j.plipres.2020.101031 32339554 PMC8350976

[108] Di Pietro P Izzo C Abate AC Iesu P Rusciano MR Venturini E The dark side of sphingolipids: searching for potential cardiovascular biomarkers. Biomolecules. (2023) 13(1):168. 10.3390/biom13010168 36671552 PMC9855992

[109] Yu Z Peng Q Huang Y. Potential therapeutic targets for atherosclerosis in sphingolipid metabolism. Clin Sci. (2019) 133(6):763–76. 10.1042/CS20180911 PMC6422862 30890654

[110] WHO. The Global Health Observatory-Blood pressure/hypertension. Available at: https://www.who.int/data/gho/indicator-metadata-registry/imr-details/3155 (Accessed May 1, 2023).

[111] Bréart B Ramos-Perez WD Mendoza A Salous AK Gobert M Huang Y Lipid phosphate phosphatase 3 enables efficient thymic egress. J Exp Med. (2011) 208(6):1267–78. 10.1084/jem.20102551 21576386 PMC3173249

[112] Ikeda Y Ohashi K Shibata R Pimentel DR Kihara S Ouchi N Cyclooxygenase-2 induction by adiponectin is regulated by a sphingosine kinase-1 dependent mechanism in cardiac myocytes. FEBS Lett. (2008) 582(7):1147–50. 10.1016/j.febslet.2008.03.002 18339320 PMC2423200

[113] Jenkins RW Canals D Hannun YA. Roles and regulation of secretory and lysosomal acid sphingomyelinase. Cell Signal. (2009) 21(6):836–46. 10.1016/j.cellsig.2009.01.026 19385042 PMC3488588

[114] Kott M Elke G Reinicke M Winoto-Morbach S Schädler D Zick G Acid sphingomyelinase Serum activity predicts mortality in intensive care unit patients after systemic inflammation: a prospective cohort study. PLoS One. (2014) 9(11):e112323. 10.1371/journal.pone.0112323 25384060 PMC4226549

[115] Zietzer A Düsing P Reese L Nickenig G Jansen F. Ceramide metabolism in cardiovascular disease: a network with high therapeutic potential. Arterioscler Thromb Vasc Biol. (2022) 42(10):1220–8. 10.1161/ATVBAHA.122.318048 36004640

[116] Spijkers LJA van den Akker RFP Janssen BJA Debets JJ De Mey JGR Stroes ESG Hypertension is associated with marked alterations in sphingolipid biology: a potential role for ceramide. PLoS One. 2011;6(7):e21817. 10.1371/journal.pone.0021817 21818267 PMC3139577

[117] Di Pietro P Carrizzo A Sommella E Oliveti M Iacoviello L Di Castelnuovo A Targeting the ASMase/S1P pathway protects from sortilin-evoked vascular damage in hypertension. J Clin Invest. (2022) 132(3):e146343. 10.1172/JCI146343 35104805 PMC8803332

[118] Siedlinski M Nosalski R Szczepaniak P Ludwig-Gałęzowska AH Mikołajczyk T Filip M Vascular transcriptome profiling identifies sphingosine kinase 1 as a modulator of angiotensin II-induced vascular dysfunction. Sci Rep. (2017) 7(1):44131. 10.1038/srep44131 28276483 PMC5343497

[119] Wu X Xu J Li X Dai J Wang L. Inhibition of SphK1/S1P signaling pathway alleviates fibrosis and inflammation of rat myocardium after myocardial infarction. Comput Math Methods Med. (2022) 2022:1–11. 10.1155/2022/5985375 PMC9300330 35872958

[120] Jujic A Matthes F Vanherle L Petzka H Orho-Melander M Nilsson PM Plasma S1P (sphingosine-1-phosphate) links to hypertension and biomarkers of inflammation and cardiovascular disease: findings from a translational investigation. Hypertension. (2021) 78(1):195–209. 10.1161/HYPERTENSIONAHA.120.17379 33993723

[121] Yogi A Callera GE Aranha AB Antunes TT Graham D McBride M Sphingosine-1-Phosphate-induced inflammation involves receptor tyrosine kinase transactivation in vascular cells. Hypertension. (2011) 57(4):809–18. 10.1161/HYPERTENSIONAHA.110.162719 21383307

[122] Guzik TJ Hoch NE Brown KA McCann LA Rahman A Dikalov S Role of the T cell in the genesis of angiotensin II–induced hypertension and vascular dysfunction. J Exp Med. (2007) 204(10):2449–60. 10.1084/jem.20070657 17875676 PMC2118469

[123] Obinata H Hla T. Sphingosine 1-phosphate and inflammation. Int Immunol. (2019) 31(9):617–25. 10.1093/intimm/dxz037 31049553 PMC6939830

[124] Aoki M Aoki H Ramanathan R Hait NC Takabe K. Sphingosine-1-phosphate signaling in immune cells and inflammation: roles and therapeutic potential. Mediators Inflamm. (2016) 2016:1–11. 10.1155/2016/8606878 PMC4761394 26966342

[125] Meissner A Miro F Jiménez-Altayó F Jurado A Vila E Planas AM. Sphingosine-1-phosphate signalling—a key player in the pathogenesis of angiotensin II-induced hypertension. Cardiovasc Res. (2017) 113(2):123–33. 10.1093/cvr/cvw256 28082452

[126] Yalcinkaya M Fotakis P Liu W Endo-Umeda K Dou H Abramowicz S Cholesterol accumulation in macrophages drives NETosis in atherosclerotic plaques via IL-1β secretion. Cardiovasc Res. (2023) 119(4):969–81. 10.1093/cvr/cvac189 36537208 PMC10153645

[127] Ding L Biswas S Morton RE Smith JD Hay N Byzova TV Akt3 deficiency in macrophages promotes foam cell formation and atherosclerosis in mice. Cell Metab. (2012) 15(6):861–72. 10.1016/j.cmet.2012.04.020 22632897 PMC3372639

[128] Barrett TJ. Macrophages in atherosclerosis regression. Arterioscler Thromb Vasc Biol. (2020) 40(1):20–33. 10.1161/ATVBAHA.119.312802 31722535 PMC6946104

[129] Xu H Jiang J Chen W Li W Chen Z. Vascular macrophages in atherosclerosis. J Immunol Res. (2019) 2019:1–14. 10.1155/2019/4354786 PMC6914912 31886303

[130] Alewijnse AE Peters SLM. Sphingolipid signalling in the cardiovascular system: good, bad or both? Eur J Pharmacol. (2008) 585(2–3):292–302. 10.1016/j.ejphar.2008.02.089 18420192

[131] Ruiz M Frej C Holmér A Guo LJ Tran S Dahlbäck B. High-density lipoprotein–associated apolipoprotein M limits endothelial inflammation by delivering sphingosine-1-phosphate to the sphingosine-1-phosphate receptor 1. Arterioscler Thromb Vasc Biol. (2017) 37(1):118–29. 10.1161/ATVBAHA.116.308435 27879252

[132] Sanna MG Liao J Jo E Alfonso C Ahn MY Peterson MS Sphingosine 1-phosphate (S1P) receptor subtypes S1P1 and S1P3, respectively, regulate lymphocyte recirculation and heart rate. J Biol Chem. (2004) 279(14):13839–48. 10.1074/jbc.M311743200 14732717

[133] Galvani S Sanson M Blaho VA Swendeman SL Obinata H Conger H HDL-bound sphingosine 1-phosphate acts as a biased agonist for the endothelial cell receptor S1P 1 to limit vascular inflammation. Sci Signal. (2015) 8(389):ra79. 10.1126/scisignal.aaa2581 26268607 PMC4768813

[134] Keul P Polzin A Kaiser K Gräler M Dannenberg L Daum G Potent anti-inflammatory properties of HDL in vascular smooth muscle cells mediated by HDL-S1P and their impairment in coronary artery disease due to lower HDL-S1P: a new aspect of HDL dysfunction and its therapy. FASEB J. (2019) 33(1):1482–95. 10.1096/fj.201801245R 30130432

[135] Feuerborn R Becker S Potì F Nagel P Brodde M Schmidt H High density lipoprotein (HDL)-associated sphingosine 1-phosphate (S1P) inhibits macrophage apoptosis by stimulating STAT3 activity and survivin expression. Atherosclerosis. (2017) 257:29–37. 10.1016/j.atherosclerosis.2016.12.009 28038379

[136] Gonzalez L Qian A Tahir U Yu P Trigatti B. Sphingosine-1-Phosphate receptor 1, expressed in myeloid cells, slows diet-induced atherosclerosis and protects against macrophage apoptosis in ldlr KO mice. Int J Mol Sci. (2017) 18(12):2721. 10.3390/ijms18122721 29244772 PMC5751322

[137] Weis T Völker W Holtwick R Al Chahaf M Schmidt A. Sphingosine 1-phosphate (S1P) induces expression of E-selectin and adhesion of monocytes via intracellular signalling pathways in vascular endothelial cells. Eur J Cell Biol. (2010) 89(10):733–41. 10.1016/j.ejcb.2010.06.011 20656374

[138] Singh RK Haka AS Brumfield A Grosheva I Bhardwaj P Chin HF Ceramide activation of RhoA/rho kinase impairs actin polymerization during aggregated LDL catabolism. J Lipid Res. (2017) 58(10):1977–87. 10.1194/jlr.M076398 28814641 PMC5625121

[139] Laulederkind SJ Bielawska A Raghow R Hannun YA Ballou LR. Ceramide induces interleukin 6 gene expression in human fibroblasts. J Exp Med. (1995) 182(2):599–604. 10.1084/jem.182.2.599 7629516 PMC2192147

[140] Augé N Nègre-Salvayre A Salvayre R Levade T. Sphingomyelin metabolites in vascular cell signaling and atherogenesis. Prog Lipid Res. (2000) 39(3):207–29. 10.1016/S0163-7827(00)00007-2 10799716

[141] Meeusen JW Donato LJ Kopecky SL Vasile VC Jaffe AS Laaksonen R. Ceramides improve atherosclerotic cardiovascular disease risk assessment beyond standard risk factors. Clin Chim Acta. (2020) 511:138–42. 10.1016/j.cca.2020.10.005 33058843

[142] Lallemand T Rouahi M Swiader A Grazide MH Geoffre N Alayrac P nSMase2 (type 2-neutral sphingomyelinase) deficiency or inhibition by GW4869 reduces inflammation and atherosclerosis in ApoE−/− mice. Arterioscler Thromb Vasc Biol. (2018) 38(7):1479–92. 10.1161/ATVBAHA.118.311208 29794115 PMC6039418

[143] Jiang XC Paultre F Pearson TA Reed RG Francis CK Lin M Plasma sphingomyelin level as a risk factor for coronary artery disease. Arterioscler Thromb Vasc Biol. (2000) 20(12):2614–8. 10.1161/01.ATV.20.12.2614 11116061

[144] Laaksonen R Ekroos K Sysi-Aho M Hilvo M Vihervaara T Kauhanen D Plasma ceramides predict cardiovascular death in patients with stable coronary artery disease and acute coronary syndromes beyond LDL-cholesterol. Eur Heart J. (2016) 37(25):1967–76. 10.1093/eurheartj/ehw148 27125947 PMC4929378

[145] Poss AM Maschek JA Cox JE Hauner BJ Hopkins PN Hunt SC Machine learning reveals serum sphingolipids as cholesterol-independent biomarkers of coronary artery disease. J Clin Invest. (2020) 130(3):1363–76. 10.1172/JCI131838 31743112 PMC7269567

[146] Zhu D Mackenzie NCW Farquharson C MacRae VE. Mechanisms and clinical consequences of vascular calcification. Front Endocrinol (Lausanne). (2012) 3:28219. 10.3389/fendo.2012.00095 PMC3412412 22888324

[147] Jain S Khera R Corrales–Medina VF Townsend RR Chirinos JA. “Inflammation and arterial stiffness in humans”. Atherosclerosis. (2014) 237(2):381–90. 10.1016/j.atherosclerosis.2014.09.011 25463062

[148] Laurent S Boutouyrie P. Arterial stiffness and hypertension in the elderly. Front Cardiovasc Med. (2020) 7:544302. 10.3389/fcvm.2020.544302 33330638 PMC7673379

[149] McEniery CM McDonnell BJ So A Aitken S Bolton CE Munnery M Aortic calcification is associated with aortic stiffness and isolated systolic hypertension in healthy individuals. Hypertension. (2009) 53(3):524–31. 10.1161/HYPERTENSIONAHA.108.126615 19171791

[150] Durham AL Speer MY Scatena M Giachelli CM Shanahan CM. Role of smooth muscle cells in vascular calcification: implications in atherosclerosis and arterial stiffness. Cardiovasc Res. (2018) 114(4):590–600. 10.1093/cvr/cvy010 29514202 PMC5852633

[151] Chen NX Moe SM. Vascular calcification: pathophysiology and risk factors. Curr Hypertens Rep. (2012) 14(3):228–37. 10.1007/s11906-012-0265-8 22476974 PMC3959826

[152] Mattace-Raso FUS van der Cammen TJM Hofman A van Popele NM Bos ML Schalekamp MADH Arterial stiffness and risk of coronary heart disease and stroke. Circulation. (2006) 113(5):657–63. 10.1161/CIRCULATIONAHA.105.555235 16461838

[153] Oliver JJ Webb DJ. Noninvasive assessment of arterial stiffness and risk of atherosclerotic events. Arterioscler Thromb Vasc Biol. (2003) 23(4):554–66. 10.1161/01.ATV.0000060460.52916.D6 12615661

[154] Takuwa N Ohkura SI Takashima SI Ohtani K Okamoto Y Tanaka T S1P3-mediated cardiac fibrosis in sphingosine kinase 1 transgenic mice involves reactive oxygen species. Cardiovasc Res. (2010) 85(3):484–93. 10.1093/cvr/cvp312 19755413 PMC2802201

[155] Morris TG Borland SJ Clarke CJ Wilson C Hannun YA Ohanian V Sphingosine 1-phosphate activation of ERM contributes to vascular calcification. J Lipid Res. (2018) 59(1):69–78. 10.1194/jlr.M079731 29167409 PMC5748498

[156] Luong TTD Tuffaha R Schuchardt M Moser B Schelski N Boehme B Acid sphingomyelinase promotes SGK1-dependent vascular calcification. Clin Sci. (2021) 135(3):515–34. 10.1042/CS20201122 PMC7859357 33479769

[157] Bhat OM Li G Yuan X Huang D Gulbins E Kukreja RC Arterial medial calcification through enhanced small extracellular vesicle release in smooth muscle-specific Asah1 gene knockout mice. Sci Rep. (2020) 10(1):1645. 10.1038/s41598-020-58568-5 32015399 PMC6997457

[158] Bhat OM Yuan X Cain C Salloum FN Li P. Medial calcification in the arterial wall of smooth muscle cell-specific *Smpd1* transgenic mice: a ceramide-mediated vasculopathy. J Cell Mol Med. (2020) 24(1):539–53. 10.1111/jcmm.14761 31743567 PMC6933411

[159] Liao L Zhou Q Song Y Wu W Yu H Wang S Ceramide mediates ox-LDL-induced human vascular smooth muscle cell calcification via p38 mitogen-activated protein kinase signaling. PLoS One. (2013) 8(12):e82379. 10.1371/journal.pone.0082379 24358176 PMC3865066

[160] Habibi J DeMarco VG Hulse JL Hayden MR Whaley-Connell A Hill MA Inhibition of sphingomyelinase attenuates diet—induced increases in aortic stiffness. J Mol Cell Cardiol. (2022) 167:32–9. 10.1016/j.yjmcc.2022.03.006 35331697 PMC9107502

[161] Li C He J Li S Chen W Bazzano L Sun X Novel metabolites are associated with augmentation index and pulse wave velocity: findings from the bogalusa heart study. Am J Hypertens. (2019) 32(6):547–56. 10.1093/ajh/hpz046 30953049 PMC6508455

[162] Chatterjee S Bedja D Mishra S Amuzie C Avolio A Kass DA Inhibition of glycosphingolipid synthesis ameliorates atherosclerosis and arterial stiffness in apolipoprotein E−/− mice and rabbits fed a high-fat and -cholesterol diet. Circulation. (2014) 129(23):2403–13. 10.1161/CIRCULATIONAHA.113.007559 24710030 PMC4053506

[163] Jung S Kim M Lee YJ Lee SH Lee JH. Associations between metabolomic-identified changes of biomarkers and arterial stiffness in subjects progressing to impaired fasting glucose. Clin Endocrinol (Oxf). (2015) 83(2):196–204. 10.1111/cen.12821 25990250

[164] Sun N Keep RF Hua Y Xi G. Critical role of the sphingolipid pathway in stroke: a review of current utility and potential therapeutic targets. Transl Stroke Res. (2016) 7(5):420–38. 10.1007/s12975-016-0477-3 27339463 PMC5016220

[165] Sanchez T Skoura A Wu MT Casserly B Harrington EO Hla T. Induction of vascular permeability by the sphingosine-1-phosphate receptor–2 (S1P2R) and its downstream effectors ROCK and PTEN. Arterioscler Thromb Vasc Biol. (2007) 27(6):1312–8. 10.1161/ATVBAHA.107.143735 17431187

[166] Kim GS Yang L Zhang G Zhao H Selim M McCullough LD Critical role of sphingosine-1-phosphate receptor-2 in the disruption of cerebrovascular integrity in experimental stroke. Nat Commun. (2015) 6(1):7893. 10.1038/ncomms8893 26243335 PMC4587559

[167] Pepe G Cotugno M Marracino F Giova S Capocci L Forte M Differential expression of sphingolipid metabolizing enzymes in spontaneously hypertensive rats: a possible substrate for susceptibility to brain and kidney damage. Int J Mol Sci. (2021) 22(7):3796. 10.3390/ijms22073796 33917593 PMC8038804

[168] Lee TH Cheng CN Chao HC Lee CH Kuo CH Tang SC Plasma ceramides are associated with outcomes in acute ischemic stroke patients. J Formos Med Assoc. (2022) 121(1):43–50. 10.1016/j.jfma.2021.01.006 33504464

[169] Caughlin S Hepburn J Liu Q Wang L Yeung KKC Cechetto DF Chloroquine restores ganglioside homeostasis and improves pathological and behavioral outcomes post-stroke in the rat. Mol Neurobiol. (2019) 56(5):3552–62. 10.1007/s12035-018-1317-0 30145786

[170] Meissner A Yang J Kroetsch JT Sauvé M Dax H Momen A Tumor necrosis factor-α–mediated downregulation of the cystic fibrosis transmembrane conductance regulator drives pathological sphingosine-1-phosphate signaling in a mouse model of heart failure. Circulation. (2012) 125(22):2739–50. 10.1161/CIRCULATIONAHA.111.047316 22534621

[171] Pérez-Carrillo L Giménez-Escamilla I Martínez-Dolz L Sánchez-Lázaro IJ Portolés M Roselló-Lletí E Implication of sphingolipid metabolism gene dysregulation and cardiac sphingosine-1-phosphate accumulation in heart failure. Biomedicines. (2022) 10(1):135. 10.3390/biomedicines10010135 35052814 PMC8773611

[172] Ji R Akashi H Drosatos K Liao X Jiang H Kennel PJ Increased de novo ceramide synthesis and accumulation in failing myocardium. JCI Insight. (2017) 2(9):e82922. 28469091 10.1172/jci.insight.82922 PMC5414571

[173] Lemaitre RN Jensen PN Hoofnagle A McKnight B Fretts AM King IB Plasma ceramides and sphingomyelins in relation to heart failure risk. Circ Heart Fail. (2019) 12(7):e005708. 10.1161/CIRCHEARTFAILURE.118.005708 31296099 PMC6629465

[174] Feuerborn R Besser M Potì F Burkhardt R Weißen-Plenz G Ceglarek U Elevating endogenous sphingosine-1-phosphate (S1P) levels improves endothelial function and ameliorates atherosclerosis in low density lipoprotein receptor-deficient (LDL-R−/−) mice. Thromb Haemost. (2018) 118(08):1470–80. 10.1055/s-0038-1666870 30060257

[175] Polzin A Piayda K Keul P Dannenberg L Mohring A Gräler M Plasma sphingosine-1-phosphate concentrations are associated with systolic heart failure in patients with ischemic heart disease. J Mol Cell Cardiol. (2017) 110:35–7. 10.1016/j.yjmcc.2017.07.004 28709768

[176] Gowda SGB Gowda D Kain V Chiba H Hui SP Chalfant CE Sphingosine-1-phosphate interactions in the spleen and heart reflect extent of cardiac repair in mice and failing human hearts. Am J Physiol Heart Circ Physiol. (2021) 321(3):H599–611. 10.1152/ajpheart.00314.2021 34415189 PMC8461844

[177] Zhang F Xia Y Yan W Zhang H Zhou F Zhao S Sphingosine 1-phosphate signaling contributes to cardiac inflammation, dysfunction, and remodeling following myocardial infarction. Am J Physiol Heart Circ Physiol. (2016) 310(2):H250–61. 10.1152/ajpheart.00372.2015 26589326

[178] Nofer JR Bot M Brodde M Taylor PJ Salm P Brinkmann V FTY720, a synthetic sphingosine 1 phosphate analogue, inhibits development of atherosclerosis in low-density lipoprotein receptor–deficient mice. Circulation. (2007) 115(4):501–8. 10.1161/CIRCULATIONAHA.106.641407 17242282

[179] Egom EEA Ke Y Musa H Mohamed TMA Wang T Cartwright E FTY720 prevents ischemia/reperfusion injury-associated arrhythmias in an ex vivo rat heart model via activation of Pak1/Akt signaling. J Mol Cell Cardiol. (2010) 48(2):406–14. 10.1016/j.yjmcc.2009.10.009 19852968 PMC3102016

[180] Liu W Zi M Naumann R Ulm S Jin J Taglieri DM Pak1 as a novel therapeutic target for antihypertrophic treatment in the heart. Circulation. (2011) 124(24):2702–15. 10.1161/CIRCULATIONAHA.111.048785 22082674 PMC3242076

[181] Cantalupo A Zhang Y Kothiya M Galvani S Obinata H Bucci M Nogo-B regulates endothelial sphingolipid homeostasis to control vascular function and blood pressure. Nat Med. (2015) 21(9):1028–37. 10.1038/nm.3934 26301690 PMC4692717

[182] Józefczuk E Nosalski R Saju B Crespo E Szczepaniak P Guzik TJ Cardiovascular effects of pharmacological targeting of sphingosine kinase 1. Hypertension. (2020) 75(2):383–92. 10.1161/HYPERTENSIONAHA.119.13450 31838904 PMC7055939

[183] Ji Y Chen J Pang L Chen C Ye J Liu H The acid sphingomyelinase inhibitor amitriptyline ameliorates TNF-α-induced endothelial dysfunction. Cardiovasc Drugs Ther. (2022). 10.1007/s10557-022-07378-0 PMC10876840 36103099

[184] Leger AJ Mosquea LM Li L Chuang W Pacheco J Taylor K Adeno-associated virus-mediated expression of acid sphingomyelinase decreases atherosclerotic lesion formation in apolipoprotein E^−/−^ mice. J Gene Med. (2011) 13(6):324–32. 10.1002/jgm.1575 21674735

[185] Park TS Rosebury W Kindt EK Kowala MC Panek RL. Serine palmitoyltransferase inhibitor myriocin induces the regression of atherosclerotic plaques in hyperlipidemic ApoE-deficient mice. Pharmacol Res. (2008) 58(1):45–51. 10.1016/j.phrs.2008.06.005 18611440

[186] Chatterjee S Bedja D Mishra S Amuzie C Avolio A Kass DA Inhibition of glycosphingolipid synthesis ameliorates atherosclerosis and arterial stiffness in apolipoprotein E^−/−^ mice and rabbits fed a high-fat and -cholesterol diet. Circulation. (2014) 129(23):2403–13. 10.1161/CIRCULATIONAHA.113.007559 24710030 PMC4053506

[187] Savira F Magaye R Scullino CV Flynn BL Pitson SM Anderson D Sphingolipid imbalance and inflammatory effects induced by uremic toxins in heart and kidney cells are reversed by dihydroceramide desaturase 1 inhibition. Toxicol Lett. (2021) 350:133–42. 10.1016/j.toxlet.2021.07.012 34303789

[188] Koh IU Jun HS Choi JS Lim JH Kim WH Yoon JB Fenretinide ameliorates insulin resistance and fatty liver in obese mice. Biol Pharm Bull. (2012) 35(3):369–75. 10.1248/bpb.35.369 22382323

[189] Harris CM Mittelstadt S Banfor P Bousquet P Duignan DB Gintant G Sphingosine-1-phosphate (S1P) lyase inhibition causes increased cardiac S1P levels and bradycardia in rats. J Pharmacol Exp Ther. (2016) 359(1):151–8. 10.1124/jpet.116.235002 27519818

[190] Chun J Giovannoni G Hunter SF. Sphingosine 1-phosphate receptor modulator therapy for multiple sclerosis: differential downstream receptor signalling and clinical profile effects. Drugs. (2021) 81(2):207–31. 10.1007/s40265-020-01431-8 33289881 PMC7932974

[191] Sharma S Mathur AG Pradhan S Singh DB Gupta S. Fingolimod (FTY720): first approved oral therapy for multiple sclerosis. J Pharmacol Pharmacother. (2011) 2(1):49–51. 10.4103/0976-500X.77118 21701650 PMC3117573

[192] Imeri F Blanchard O Jenni A Schwalm S Wünsche C Zivkovic A FTY720 and two novel butterfly derivatives exert a general anti-inflammatory potential by reducing immune cell adhesion to endothelial cells through activation of S1P3 and phosphoinositide 3-kinase. Naunyn Schmiedebergs Arch Pharmacol. (2015) 388(12):1283–92. 10.1007/s00210-015-1159-5 26267293

[193] Lublin F Miller DH Freedman MS Cree BAC Wolinsky JS Weiner H Oral fingolimod in primary progressive multiple sclerosis (INFORMS): a phase 3, randomised, double-blind, placebo-controlled trial. Lancet. (2016) 387(10023):1075–84. 10.1016/S0140-6736(15)01314-8 26827074

[194] Camm J Hla T Bakshi R Brinkmann V. Cardiac and vascular effects of fingolimod: mechanistic basis and clinical implications. Am Heart J. (2014) 168(5):632–44. 10.1016/j.ahj.2014.06.028 25440790

[195] Harada T Wilbraham D La Borderie G Inoue S Bush J Camm AJ. Cardiac effects of amiselimod compared with fingolimod and placebo: results of a randomised, parallel-group, phase I study in healthy subjects. Br J Clin Pharmacol. (2017) 83(5):1011–27. 10.1111/bcp.13203 27921320 PMC5401982

[196] Wexler M. Amiselimod for multiple sclerosis (2022).

[197] Hughes JE Srinivasan S Lynch KR Proia RL Ferdek P Hedrick CC. Sphingosine-1-phosphate induces an antiinflammatory phenotype in macrophages. Circ Res. (2008) 102(8):950–8. 10.1161/CIRCRESAHA.107.170779 18323526 PMC2875063

[198] Gollmann G Neuwirt H Tripp CH Mueller H Konwalinka G Heufler C Sphingosine-1-phosphate receptor type-1 agonism impairs blood dendritic cell chemotaxis and skin dendritic cell migration to lymph nodes under inflammatory conditions. Int Immunol. (2008) 20(7):911–23. 10.1093/intimm/dxn050 18495625

[199] Karuppuchamy T Tyler CJ Lundborg LR Pérez-Jeldres T Kimball AK Clambey ET Sphingosine-1-phosphate lyase inhibition alters the S1P gradient and ameliorates crohn’s-like ileitis by suppressing thymocyte maturation. Inflamm Bowel Dis. (2020) 26(2):216–28. 10.1093/ibd/izz174 31807751 PMC6943703

[200] Yi X Tang X Li T Chen L He H Wu X Therapeutic potential of the sphingosine kinase 1 inhibitor, PF-543. Biomed Pharmacother. (2023);163:114401. 10.1016/j.biopha.2023.114401 37167721

[201] Shao JJ Peng Y Wang LM Wang JK Chen X. Activation of SphK1 by K6PC-5 inhibits oxygen–glucose deprivation/reoxygenation-induced myocardial cell death. DNA Cell Biol. (2015);34(11):669–76. 10.1089/dna.2015.2959 26308910 PMC4642826

[202] Potì F Ceglarek U Burkhardt R Simoni M Nofer JR. SKI-II—a sphingosine kinase 1 inhibitor—exacerbates atherosclerosis in low-density lipoprotein receptor-deficient (LDL-R−/−) mice on high cholesterol diet. Atherosclerosis. (2015) 240(1):212–5. 10.1016/j.atherosclerosis.2015.03.020 25801013

[203] Chen B Collen LV Mowat C Isaacs KL Singh S Kane SV Inflammatory bowel disease and cardiovascular diseases. Am J Med. (2022) 135(12):1453–60. 10.1016/j.amjmed.2022.08.012 36058305

[204] Maines LW Fitzpatrick LR French KJ Zhuang Y Xia Z Keller SN Suppression of ulcerative colitis in mice by orally available inhibitors of sphingosine kinase. Dig Dis Sci. (2008) 53(4):997–1012. 10.1007/s10620-007-0133-6 18058233 PMC2660406

[205] Ishimaru K Yoshioka K Kano K Kurano M Saigusa D Aoki J Sphingosine kinase-2 prevents macrophage cholesterol accumulation and atherosclerosis by stimulating autophagic lipid degradation. Sci Rep. (2019) 9(1):18329. 10.1038/s41598-019-54877-6 31797978 PMC6892873

[206] Simonis A Schubert-Unkmeir A. The role of acid sphingomyelinase and modulation of sphingolipid metabolism in bacterial infection. Biol Chem. (2018) 399(10):1135–46. 10.1515/hsz-2018-0200 29924727

[207] Devlin CM Leventhal AR Kuriakose G Schuchman EH Williams KJ Tabas I. Acid sphingomyelinase promotes lipoprotein retention within early atheromata and accelerates lesion progression. Arterioscler Thromb Vasc Biol. (2008) 28(10):1723–30. 10.1161/ATVBAHA.108.173344 18669882 PMC2562252

[208] Hojjati MR Li Z Zhou H Tang S Huan C Ooi E Effect of myriocin on plasma sphingolipid metabolism and atherosclerosis in ApoE-deficient mice. J Biol Chem. (2005) 280(11):10284–9. 10.1074/jbc.M412348200 15590644

[209] Caretti A Bragonzi A Facchini M De Fino I Riva C Gasco P Anti-inflammatory action of lipid nanocarrier-delivered myriocin: therapeutic potential in cystic fibrosis. Biochim Biophys Acta. (2014) 1840(1):586–94. 10.1016/j.bbagen.2013.10.018 24141140 PMC4097882

[210] Reforgiato MR Milano G Fabriàs G Casas J Gasco P Paroni R Inhibition of ceramide de novo synthesis as a postischemic strategy to reduce myocardial reperfusion injury. Basic Res Cardiol. (2016) 111(2):12. 10.1007/s00395-016-0533-x 26786259

[211] Yu Z Peng Q Li S Hao H Deng J Meng L Myriocin and D-PDMP ameliorate atherosclerosis in ApoE−/− mice via reducing lipid uptake and vascular inflammation. Clin Sci. (2020) 134(5):439–58. 10.1042/CS20191028 32091078

[212] Baccam GC Xie J Jin X Park H Wang B Husson H Glucosylceramide synthase inhibition protects against cardiac hypertrophy in chronic kidney disease. Sci Rep. (2022) 12(1):9340. 10.1038/s41598-022-13390-z 35660779 PMC9167280

[213] Wang X Dong J Zhao Y Li Y Wu M. Adenovirus-mediated sphingomyelin synthase 2 increases atherosclerotic lesions in ApoE KO mice. Lipids Health Dis. (2011) 10(1):7. 10.1186/1476-511X-10-7 21235823 PMC3032723

[214] Liu J Huan C Chakraborty M Zhang H Lu D Kuo MS Macrophage sphingomyelin synthase 2 deficiency decreases atherosclerosis in mice. Circ Res. (2009) 105(3):295–303. 10.1161/CIRCRESAHA.109.194613 19590047 PMC2746935

[215] Mo M Yang J Jiang XC Cao Y Fei J Chen Y Discovery of 4-benzyloxybenzo[*d* ]isoxazole-3-amine derivatives as highly selective and orally efficacious human sphingomyelin synthase 2 inhibitors that reduce chronic inflammation in *db/db* mice. J Med Chem. (2018) 61(18):8241–54. 10.1021/acs.jmedchem.8b00727 30074791

[216] Russo SB Baicu CF Van Laer A Geng T Kasiganesan H Zile MR Ceramide synthase 5 mediates lipid-induced autophagy and hypertrophy in cardiomyocytes. J Clin Invest. (2012) 122(11):3919–30. 10.1172/JCI63888 23023704 PMC3484448

[217] Leonardini A D’Oria R Incalza MA Caccioppoli C Andrulli Buccheri V Cignarelli A GLP-1 receptor activation inhibits palmitate-induced apoptosis via ceramide in human cardiac progenitor cells. J Clin Endocrinol Metab. (2017) 102(11):4136–47. 10.1210/jc.2017-00970 28938428

[218] Turpin SM Nicholls HT Willmes DM Mourier A Brodesser S Wunderlich CM Obesity-induced CerS6-dependent C16:0 ceramide production promotes weight gain and glucose intolerance. Cell Metab. (2014) 20(4):678–86. 10.1016/j.cmet.2014.08.002 25295788

[219] Siddique MM Li Y Chaurasia B Kaddai VA Summers SA. Dihydroceramides: from bit players to lead actors. J Biol Chem. (2015) 290(25):15371–9. 10.1074/jbc.R115.653204 25947377 PMC4505450

[220] Zhang QJ Holland WL Wilson L Tanner JM Kearns D Cahoon JM Ceramide mediates vascular dysfunction in diet-induced obesity by PP2A-mediated dephosphorylation of the eNOS-Akt complex. Diabetes. (2012) 61(7):1848–59. 10.2337/db11-1399 22586587 PMC3379648

[221] Lin CH Lee SY Zhang CC Du YF Hung HC Wu HT Fenretinide inhibits macrophage inflammatory mediators and controls hypertension in spontaneously hypertensive rats via the peroxisome proliferator-activated receptor gamma pathway. Drug Des Devel Ther. (2016) 10:3591–7. 10.2147/DDDT.S114879 27843299 PMC5098527

[222] Aragón-Herrera A Feijóo-Bandín S Otero Santiago M Barral L Campos-Toimil M Gil-Longo J Empagliflozin reduces the levels of CD36 and cardiotoxic lipids while improving autophagy in the hearts of zucker diabetic fatty rats. Biochem Pharmacol. (2019) 170:113677. 10.1016/j.bcp.2019.113677 31647926

[223] Voors AA Angermann CE Teerlink JR Collins SP Kosiborod M Biegus J The SGLT2 inhibitor empagliflozin in patients hospitalized for acute heart failure: a multinational randomized trial. Nat Med. (2022) 28(3):568–74. 10.1038/s41591-021-01659-1 35228754 PMC8938265

[224] Anker SD Butler J Filippatos G Ferreira JP Bocchi E Böhm M Empagliflozin in heart failure with a preserved ejection fraction. N Engl J Med. (2021) 385(16):1451–61. 10.1056/NEJMoa2107038 34449189

[225] Amini M Zayeri F Salehi M. Trend analysis of cardiovascular disease mortality, incidence, and mortality-to-incidence ratio: results from global burden of disease study 2017. BMC Public Health. (2021) 21(1):401. 10.1186/s12889-021-10429-0 33632204 PMC7905904

[226] Henein MY Vancheri S Longo G Vancheri F. The role of inflammation in cardiovascular disease. Int J Mol Sci. (2022) 23(21):12906. 10.3390/ijms232112906 36361701 PMC9658900

[227] Frąk W Wojtasińska A Lisińska W Młynarska E Franczyk B Rysz J. Pathophysiology of cardiovascular diseases: new insights into molecular mechanisms of atherosclerosis. Biomedicines. (2022) 10(8):1938. 36009488 10.3390/biomedicines10081938 PMC9405799

[228] Choi RH Tatum SM Symons JD Summers SA Holland WL. Ceramides and other sphingolipids as drivers of cardiovascular disease. Nat Rev Cardiol. (2021) 18(10):701–11. 10.1038/s41569-021-00536-1 33772258 PMC8978615

[229] Santos-Gallego CG Vahl TP Goliasch G Picatoste B Arias T Ishikawa K Sphingosine-1-phosphate receptor agonist fingolimod increases myocardial salvage and decreases adverse postinfarction left ventricular remodeling in a porcine model of ischemia/reperfusion. Circulation. (2016) 133(10):954–66. 10.1161/CIRCULATIONAHA.115.012427 26826180

[230] Dunnington K Benrimoh N Brandquist C Cardillo-Marricco N Di Spirito M Grenier J. Application of pharmacokinetics in early drug development. In: Malangu N, editor. Pharmacokinetics and adverse effects of drugs—mechanisms and risks factors. InTech (2018).

[231] Huggins DJ Sherman W Tidor B. Rational approaches to improving selectivity in drug design. J Med Chem. (2012) 55(4):1424–44. 10.1021/jm2010332 22239221 PMC3285144

[232] Mitchell MJ Billingsley MM Haley RM Wechsler ME Peppas NA Langer R. Engineering precision nanoparticles for drug delivery. Nat Rev Drug Discov. (2021) 20(2):101–24. 10.1038/s41573-020-0090-8 33277608 PMC7717100

[233] Tamanna RJ Alam MI Hossain A Khan MHR. On sample size calculation in testing treatment efficacy in clinical trials. Biom Lett. (2021) 58(2):133–47. 10.2478/bile-2021-0010

[234] Resnik D. Beyond post-marketing research and MedWatch: long-term studies of drug risks. Drug Des Devel Ther. (2007) 1:1–5. 10.2147/DDDT.S2352 19727333 PMC2763348

